# Functional and safety profiles of microbial communities in milk of ranched and nomadic goats and their predictive role in mycotoxin reduction

**DOI:** 10.1007/s11274-025-04507-3

**Published:** 2025-08-01

**Authors:** Muiz O. Akinyemi, Mariska S. Kleyn, Mona Abdelmaksoud, Deidré A. B. Van Wyk, Rasheed A. Adeleke, Chibundu N. Ezekiel

**Affiliations:** 1 Leeds Institute of Health Sciences, University of Leeds, Leeds, LS2 9NL UK; 2 Unit for Environmental Sciences and Management, North-West University, Potchefstroom, 2520 South Africa; 3 College of Health and Science, University of Lincoln, Lincoln, LN6 7DL UK; 4 Unit for Environment Science and Management, Microbiology, North-West University, Mahikeng campus, Private Bag X2046, Mahikeng, 2745 South Africa; 5 BOKU University, Institute of Bioanalytics and Agro-Metabolomics, Department of Agricultural Sciences, Konrad Lorenz Str. 20, Tulln, 3430 Austria

**Keywords:** Bacteria, Decontamination, Dairy, Mycotoxin, Pathogen, Probiotic, Yeast

## Abstract

This study investigates the microbial dynamics, mycotoxin reduction, and biotechnological potentials of bacteria and yeasts in raw goat milk from Nigeria’s ranched and nomadic farming systems. The 16S/26S rRNA gene sequencing identified a rich microbial diversity in goat milk, dominated by bacterial phyla: Firmicutes (also known as Bacillota) (86.5%), Proteobacteria (also known as Pseudomonadota) (13.2%), Actinobacteriota (0.3%), and yeast phylotypes: Ascomycota (55%) and Basidiomycota (44%). Overall, the abundance of the bacteria *Enterococcus*, *Bacillus*, *Streptococcus*, *Escherichia*-*Shigella*, and *Clostridium* sensu stricto 1, and the yeasts *Pseudotremella*, *Saccharomyces*, *Candida*, *Kluyveromyces*, and *Trichosporon* accounted for at least 90% of the total genera detected. Significant differences (*p* < 0.05) in microbial compositions were observed between ranched and nomadic goats, with the latter showing higher richness (Chao1: 59.9 vs. 41.6) and higher abundances of key genera including potential pathogenic groups such as *Bacillus*,* Klebsiella*,* Neisseria*,* Pseudomonas*,* Serratia*,* Streptococcus*, and *Cryptococcus*. Functional assays highlighted probiotic traits in lactic acid bacteria, e.g., *Limosilactobacillus fermentum 30m1* and *Weissella cibaria 93m3*, and biotechnological abilities in *Pediococcus acidilactici 18a*. Mycotoxin analysis revealed frequent contamination in critically low levels (<500 ng/L), including aflatoxin M_1_ (96.2% of samples) and citrinin (61.5%), with nomadic milk exhibiting slightly higher mycotoxin levels than milk from ranched goats. Moderate correlations (0.25 < r^2^ < 0.49) were found between certain bacterial and yeast genera, such as *Streptococcus* and *Saccharomyces*, and concentrations of mycotoxins like aflatoxin B_1_ and M_1_, suggesting a potential role for these microbes as mycotoxin decontaminators. Our results indicate that while nomadic practices enhance microbial diversity, they also pose safety risks due to increased pathogen and mycotoxin prevalences. These findings underscore the need for improved farm management to balance microbial benefits (e.g., probiotics, mycotoxin mitigation) with public health risks. This study provides actionable insights for developing safe fermented dairy products and policies to enhance food security in sub-Saharan Africa.

**Supplementary Information:**

The online version contains supplementary material available at 10.1007/s11274-025-04507-3.

## Introduction

Goat milk is a highly digestible source of essential nutrients in the human diet, offering a rich source of proteins, fats, vitamins, and minerals (Nelson et al. 2023). Its unique composition makes it particularly advantageous for vulnerable populations, such as children, the elderly, and individuals with cow milk allergies (Silanikove et al. 2010). In Nigeria, where over 90% of dairy milk is sourced from cows, there has been a surge in demand for goat milk and its derivatives due to an increased interest in alternative milk sources (Atibioke et al. 2022; Hadef et al. 2024). While most mainstream products in Nigeria are derived from the West African dwarf (or Nigerian dwarf) breed, milk from other breeds, such as the Sahel and red Sokoto, is also integral to traditional food production, especially among low-income households. Despite its nutritional benefits, the safety of goat milk in Nigeria, particularly regarding microbiological hazards and chemical contaminants such as mycotoxins, remains understudied compared to cow milk.

Beyond its nutritional value, goat milk harbours a diverse microbial community that includes both beneficial and pathogenic organisms. Lactic acid bacteria (LAB) such as *Lactobacillus*, *Lactococcus*,* Pediococcus*, and *Streptococcus* species are key contributors to milk fermentation, enhancing product safety, shelf life, and sensory attributes through the production of antimicrobial compounds like bacteriocins and organic acids (Oikonomou et al. 2020; Parente et al. 2020). Certain LAB strains also exhibit probiotic properties, linked to improved gut health, immune function, and reduced chronic disease risks (Akinyemi et al. 2022b; Rajput et al. 2022). Fermented products like yogurt and kefir further capitalize on these benefits, aligning with global trends toward functional foods. However, the microbiological quality of goat milk is vulnerable to contamination during production, handling, or storage, particularly in Nigeria’s artisanal systems (Parente et al. 2020). Dairy goats in Nigeria are raised under two primary systems: ranching and nomadic herding. Ranched goats are bred in confined spaces where food and shelter are provided, while nomadic goats forage freely in the environment surrounding their owner’s home or in nearby communities.

In many rural settings, milking is typically performed manually into containers, and the milk is often consumed raw (Hadef et al. 2024) or used immediately to produce traditional fermented food products (Ehirim and Onyeneke 2013; Igwegbe et al. 2015; Onyeke 2020). The consumption or use of raw milk for food production, poses dual risks. While it may retain beneficial antibodies and microbes (Korhonen et al. 2000), the risks associated with exposure to harmful microbes and chemical contaminants like mycotoxins far outweigh these advantages, posing significant threats to public health (Akinyemi et al. 2021). Pathogens such as *Listeria monocytogenes*, *Salmonella spp.*, *Escherichia coli* O157:H7, *Mycobacterium bovis*, and *Brucella melitensis* have been detected in milk and milk products in Nigeria, contributing to outbreaks of foodborne illnesses (Oduori et al. 2022). Mycotoxins, defined simply as toxic secondary metabolites of moulds, are of particular concern due to their carcinogenic and immunosuppressive effects (Milićević et al. 2010). However, emerging evidence suggests that certain milk microbiota, including detoxification-capable strains, may mitigate mycotoxin risks through enzymatic degradation or binding mechanisms (Gao et al. 2011; Adebo et al. 2016; Raksha Rao et al. 2017).

Despite the potential of goat milk as a vehicle for delivering beneficial microbes, the risk associated with improper handling, poor hygiene practices, and inadequate storage conditions during artisanal milk production in Nigeria remain significant challenges (Christianah and Opadoyin 2016; Oduori et al. 2022). These issues increase the risk of contamination with harmful pathogens, overshadowing the positive contributions of beneficial microbes. Moreover, limited research exists on how factors such as farming systems and goat breeds influence the balance between beneficial and pathogenic microbes in goat milk. Understanding this dynamic is essential for optimizing the safety and functionality of goat milk products. To address these gaps, this study aimed at providing a comprehensive analysis of the microbiological safety and functional profiles of goat milk microbiota in Nigeria. Specifically, the study sought to: (i) Assess the impact of farming systems on microbial diversity across three goat breeds, (ii) Evaluate virulence factors and functional traits of milk microbiota, and (iii) predict the interaction between microbiota and mycotoxins in raw milk, including the potential of beneficial microbes to detoxify contaminants. By integrating culture-dependent methods with cutting-edge culture-independent approaches, including next-generation sequencing, this study offers unique insights into the complex interplay between beneficial and pathogenic microbes in goat milk within a sub-Saharan African context. The findings not only advance our understanding of microbial dynamics but also provide actionable data to enhance food safety, mitigate public health risks, and guide evidence-based policies for the sustainable growth of Nigeria’s goat milk sector.

## Materials and methods

### Study sites and collection of goat milk samples

**Fig. 1:**
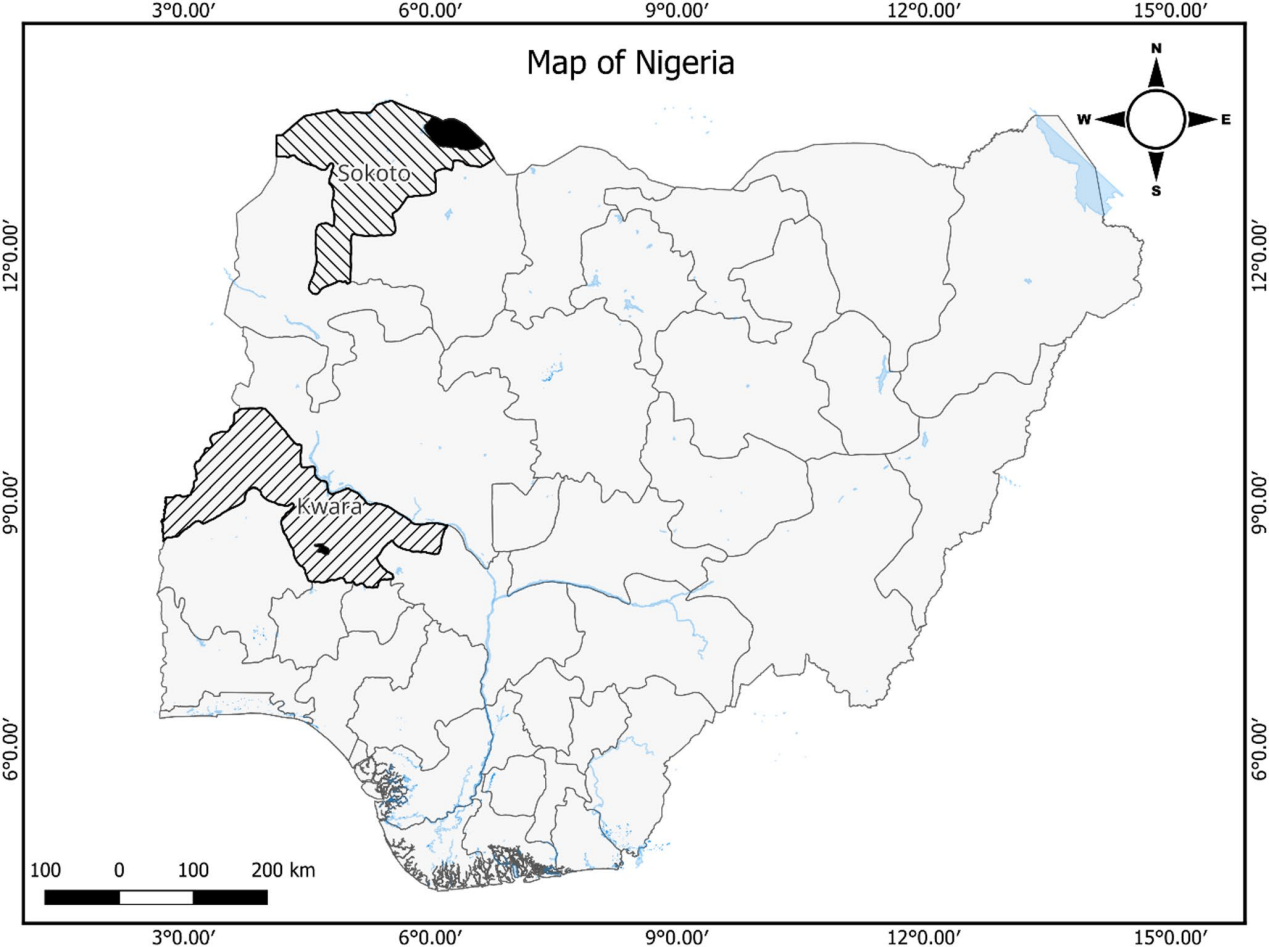
Geographic locations for goat milk sampling. Land areas filled with black stripes represent the Kwara and Sokoto states while areas filled with black shade within each state represent the local governments where the dairy farms are located

Two dairy farms in Kwara (8.9669° N, 4.3874° E) and four in Sokoto (13.0533° N, 5.3223° E) states, Nigeria (Fig. 1) were the target sites for goat milk sampling. The two states were selected due to their higher production, sale, and consumption of goat milk compared to other states in the country. The selected farms had a production scale of at least 20 dairy goats. The West African Dwarf breed was predominant in Kwara state while the Red Sokoto and Sahel goat breeds were more common in Sokoto state. All udder halves were inspected by a veterinary doctor and certified healthy, goats on antibiotic treatment were excluded from the study.

A total of 52 milk samples were collected from the dairy farms during the onset of the wet season in May 2019. The samples included ranched (*n* = 26) and nomadic (*n* = 26) goats, representing the Red Sokoto (*n* = 20), Sahel (*n* = 20), and West African Dwarf (*n* = 16) breeds. Twenty millilitres of mid-stream milk were collected from the udder of lactating goats into sterile single-use 25 mL plastic vials and transported in a cold chamber (4 °C) to the laboratory for analysis. The milk samples were collected by the owners of the dairy animals or those responsible for milking, however, to minimise the risk of contamination from the samplers’ hands, the collector wore latex gloves. Biodata including age, lactation period, and breed of each goat was retrieved from farm records where available.

Each milk sample was divided into three batches for analysis: batch A consisted of 5 mL subsamples, each dispensed into a 10 mL sterile plastic vial with tightly fitted screw cap and stored at -80 °C before DNA extraction for amplicon sequence analysis; batch B was 10 mL subsamples, each dispensed into a 50 mL Falcon tube for mycotoxin analysis; and batch C comprised of 5 mL subsamples for culture-dependent microbiological analysis involving isolation of bacteria and yeasts.

### Bacterial and yeast DNA extraction and sequencing

The samples from batch A were subjected to cold centrifugation at -20°C and 11,290 RCF for 3 min prior to removal of the supernatant fat layer. The resulting cell pellets were then resuspended and washed twice with 5 mL of 1X phosphate-buffered saline under similar centrifugation conditions. DNA extraction was performed using the Quick-DNA Faecal/Soil microbe kit (Zymo Research, Irvine, CA, United States), according to the manufacturer’s protocol, and 1 mL of cell suspension (equivalent to 1 mg). The extracted DNA was measured for quantity and quality using a nanodrop spectrophotometer (ND-one Thermo Fisher Scientific, Waltham, MA, USA). The amplicon libraries for the bacterial hypervariable V4 region of the 16S rRNA gene and D1/D2 region of the 26S rRNA gene of yeasts were prepared using the Illumina MiSeq protocol and the respective primer pairs, 515 F (5′ -GTGYCAGCMGCCGCGGTAA-3′)/806R (5′ -GGACTACNVGGGTWTCTAAT-3’) and LS2-MF (forward) (5′-GAGTCGAGTTGTTTGGGAAT-3′)/NL4 (reverse) (5′-GGTCCGTGTTTCAAGACGG-3′). Each forward and reverse primer contained Illumina overhang adapters (Illumina Inc., Foster City, CA, USA). The library preparation protocol was conducted following the steps outlined by Akinyemi et al. (2023). Precisely, the process involved initial amplicon indexing using Nextera XT primers (Illumina Inc.), and purification using AMPure XP magnetic beads. The purified libraries, along with the PhiX sequencing control, were sequenced using paired-end sequencing (2 × 300 bp) on the Illumina MiSeq platform at the Unit for Environmental Sciences and Management, North-West University, Potchefstroom, South Africa.

### Bioinformatics analysis

Microbiome bioinformatics analysis was conducted on the demultiplexed paired-end sequence reads utilising the QIIME 2 platform (Bolyen et al. 2019). The quality of the sequences was assessed using the FastQC software (Andrews, 2010), and subsequently trimmed at both the 5’- and 3’- ends to eliminate poor quality nucleotides. The sequences were denoised, merged, and depleted of chimeric sequences before clustering into Amplicon Sequence Variants (ASVs) using the DADA2 denoiser (Callahan et al. 2016) which is integrated into QIIME 2. The ASV count table resulting from this process was depleted of singletons. The taxonomic assignment of the sequences was based on a 97% identity threshold using the q2-feature classifier with a default confidence threshold of 0.7% against the SILVA v132 reference database for bacterial V3 - V4 and an in-house constructed reference database of the D1/D2 region of the partial 26 S rRNA gene for yeast. For the constructed database, 26S rRNA genes were downloaded from the National Center for Biotechnology Information (NCBI) database using the Entrez programming utility (https://www.ncbi.nlm.nih.gov/books/NBK179288/*)* in May 2021. The 26S sequences were filtered to retain only those within the phyla Ascomycota and Basidiomycota, which encompass all known yeasts, while sequences from known mould taxonomic order were discarded. A reference sequence and taxonomic classification table was generated from the filtered data and used to train a classifier using the q2-feature-classifier QIIME 2 plugin following the procedure described in Qiime2 user documentation version 2024.5 (https://docs.qiime2.org/2024.5/tutorials/feature-classifier/*).* The generated 26S reference sequence and taxonomic classification table files have been made publicly available on FigShare (links can be found in the data availability section). To ascertain the quality of taxonomic assignment, representative 26S rRNA gene sequences (about 5% of the total 26S sequences) were randomly double-checked using the BlastN search tool (< http://www.ncbi.nlm.nih.gov/blast/>). Bacterial ASVs were filtered to remove archaea, mitochondrial, and chloroplast sequences, while yeast ASVs were cleared of archaea, bacterial, and chloroplast sequences. To minimize technical noise, samples from the same farm, goat breed, and lactation stage were analysed as biological duplicates, with inconsistent ASVs (present in only one replicate) excluded. The resulting ASV tables were then imported into R software for further analysis.

### Quantification of mycotoxins in raw goat milk

The method for mycotoxin extraction and quantification from the batch B samples was according to Braun et al. (2020) as reported in detail in our previous studies where the mycotoxin levels in camel, cow and goat milk were presented (Akinyemi et al. 2022a). Precisely, milk samples were vigorously mixed with 1% formic acid-acidified acetonitrile (1 mL) for 3 min on a vortex mixer, prior to addition of 0.4 g of anhydrous magnesium sulphate and 0.1 g of sodium chloride. The mixture was then shaken, and the upper layer separated after centrifugation at 4,750*x g* for 10 min at 10 °C. The extracted sample was chilled at -20 °C overnight, centrifuged at 14,000*x g* and 4 °C, and the supernatant filtered (Chromafil Xtra PTFE filter with a 0.2 μm membrane) prior to Liquid Chromatohraphy-Tandem Mass Spectrometry (LC-MS/MS) analysis. Chromatographic separation of mycotoxin was performed on an Acquity HSS T3 column, protected by a VanGuard pre-column, with the column oven set to 40 °C and the autosampler maintained at 10 °C. A gradient elution was performed using acidified ammonium acetate in water and methanol. The analysis was conducted using an Agilent 1290 Infinity II liquid chromatography system, coupled with a Sciex QTrap^®^ 6500 + mass spectrometer equipped with a Turbo-V™ electrospray ionisation source. Data acquisition and analysis were performed using Analyst software version 1.7 and Sciex OS software version 1.6, respectively. Further details concerning the calibration curve and analyte recovery can be found in Akinyemi et al. (2022a).

### Isolation of bacteria and yeasts from goat milk samples

Subsamples (1 mL) of batch C samples were directly inoculated into broth media and incubated at 30 °C for 18 h. The media used include De-Mann Rogosa Sharpe (MRS) a selective media for isolation of lactic acid bacteria, Nutrient broth a general-purpose media, M17 for isolation of Lactococci, MRS supplemented with D-Sorbitol and MRS supplemented with vancomycin for the isolation of fastidious lactic acid bacteria, and Yeast extract peptone dextrose (YEPD) for isolation of yeasts. Each broth containing the subsamples were homogenized using a vortex mixer thereafter, 1 mL aliquot of each broth was inoculated onto agar plates corresponding to each broth using the pour plate method and incubated at 37 °C for 24–48 h. Bacterial and yeast colonies with distinct morphological features on each agar plate were sub-cultured (x2) for purification on fresh corresponding agar plates and incubated under the same conditions. Bacterial isolates were presumptively clustered into groups based on phenotypic and biochemical characteristics, including cell morphology, Gram reaction, catalase and oxidase tests, carbohydrate fermentation, gas production from glucose, and growth at 10 °C and 45 °C (data not shown). Yeasts were characterized by cell morphology and biochemical properties following taxonomic methods described by The Westerdijk Fungal Biodiversity Institute (Robert et al. 2013). All yeast and LAB isolates were stored at -20 °C in 15% YEPD glycerol broth and 15% MRS glycerol broth, respectively.

### In vitro safety and functional assessment of bacteria and yeast isolates recovered from milk samples:

### Low pH and bile salt tolerance

The tolerance of bacterial and yeast isolates to acidic conditions and bile salts was assessed following the method of Akinyemi et al. (2022b) with modifications. Cell counts from 24-hour broth cultures were standardized to *1.5 × 10*^*8*^ CFU/mL, and 1 mL of each inoculum was introduced into 9 mL of MRS broth (for bacteria) or YEPD broth (for yeast). For acid tolerance, the broths were adjusted to pH 2.5, and 5.5 (control), while for bile tolerance, broths were supplemented with 0.3% and 0.5% bile. Cell viability was determined using the pour plate method in test tubes, and growth kinetics were monitored using a bio-spectrophotometer in a 96-well microplate. Cell counts and optical density (OD_600_) readings were recorded at 3-hour intervals over a 24-hour incubation period. All experiments were performed in triplicate with the viability results expressed as the percentage survival with the formula:

$$\:Survival\left(\%\right)=\frac{{CFUml}^{-1}\left(hour\right)}{{CFUml}^{-1}\left(initial\right)}\times\:100$$

### Auto-aggregation

Bacterial and yeast cells from 20-hour broth cultures were collected by centrifugation and resuspended in phosphate buffer saline (PBS) to an optical density (OD) of 0.5 at 600 nm. The suspension was washed twice with PBS and resuspended in the same buffer. The mixture was vortexed and incubated at 37 °C without agitation, with OD measurements taken after 2 and 4 h. This assay was performed in triplicate, with five replicates per trial. Auto-aggregation percentage was calculated using the following equation:

$$\:Autoaggregation\%=1-\left(\frac{{OD}_{final}}{{OD}_{0}}\right)\times\:100$$

### Microbial adhesion to hydrocarbons (MATH)

Bacterial and yeast cells were grown in MRS and YEPD broths, respectively. Each culture was centrifuged at 3000 g for 15 min at 4 °C, and the pellet was washed twice and resuspended in PBS and standardised to an OD of 0.5 at 600 nm. Equal volumes (500 mL) of the microbial suspension and hydrocarbon (toluene) were mixed in a falcon tube and vortexed for 1 min to allow interaction. After phase separation (typically 15 min at room temperature), the aqueous phase OD_600_ was measured. All experiments were performed in triplicate. Adhesion was quantified as the percentage decrease in OD_600_ (A) relative to the initial suspension (A_0_) using:

$$\:\%Hydrophobicity=\frac{(1-A)}{{A}_{0}}\times\:\:100$$

### In vitro characterization of virulence factors of bacteria and yeast isolates recovered from raw goat milk

The enzymatic and siderophore-production activities of bacterial and yeast isolates were assessed using specific agar-based assays as follows: DNAse activity was evaluated on DNAse agar base (HiMedia, India). Isolates were streaked onto the agar surface and incubated aerobically at 37 °C for 24–48 h. Post-incubation, plates were flooded with 1 N hydrochloric acid for 5 min. Clear zones around colonies indicated DNA degradation, while opaque regions denoted negative activity. *Staphylococcus aureus* ATCC 25923 and *Escherichia coli* ATCC 25922 served as positive and negative controls, respectively.

Columbia agar base supplemented with 7% defibrinated sheep blood (Thermo Scientific, USA) was used to determine haemolytic activity. Isolates were streaked and incubated at 37 °C for 24–48 h under aerobic conditions. Haemolysis was classified as β-haemolysis (clear zones), α-haemolysis (greenish discoloration), or γ-haemolysis (no change). *Streptococcus pneumoniae* ATCC 49619 (α-haemolytic) and *S. aureus* ATCC 25923 (β-haemolytic) were used as controls.

Lipase production was tested on egg yolk agar (HiMedia, India). Spot-inoculated plates were incubated at 37 °C for 72 h. A visible opaque halo around colonies indicated lipase-mediated hydrolysis of egg yolk lipids. *Bacillus subtilis* ATCC 6633 (positive) and *E. coli* ATCC 25922 (negative) were employed as controls.

Proteolysis was assessed on skimmed milk agar (1.5% agar, 10% skim milk). Isolates were streaked and incubated at 37 °C for 48–72 h. Clear zones around colonies, resulting from casein hydrolysis, were measured (mm) using callipers. *Pseudomonas aeruginosa* ATCC 27853 (positive) and *Klebsiella pneumoniae* ATCC 700603 (negative) served as controls.

Chrome Azurol S (CAS) agar used to identify siderophore production was prepared as described by (Louden et al. 2011) Plates were spot-inoculated and incubated at 28 °C for 7 days. Siderophore production was indicated by a color change from blue to orange-yellow around colonies. *Pseudomonas putida* KT2440 (positive) and *E. coli* ATCC 25922 (negative) were used for validation.

The ability of strains that were identified as non-pathogenic to produce acids from fructose, glucose, lactose, maltose, mannitol, ribose, sucrose was determined using methods described by Cappuccino and Welsh (2017).

### Molecular characterisation of bacteria and yeast strains

Total genomic DNA from bacterial and yeast isolates was extracted using the Quick-DNA Fecal/Soil Microbe Kit (Zymo Research, Irvine, CA, USA), following the manufacturer’s protocol. DNA concentration and purity were assessed using a nanodrop spectrophotometer (ND-one Thermo Fisher Scientific, Waltham, MA, USA). Isolates were identified via partial 16S rRNA (bacteria) and 26S rRNA (yeast) gene sequencing or whole genome sequencing (WGS), as previously described Akinyemi et al. (2022b, 2024b). For partial gene amplification, universal primers 27 F (5′-AGA GTT TGA TCC TGG CTC AG-3′) and 1492R (5′-GGT TAC CTT GTT ACG ACT T-3′) were used for bacterial 16S rRNA, while NL1 (5′-CAT ATC AAT AAG CGG AGG AAA AG-3′) and NL4 (5′-GGT CCG TGT TTC AAG ACG G-3′) targeted yeast 26S rRNA. PCR amplification was performed in a BioRad^®^ ThermoCycler (Bio-Rad, USA) under standard conditions. Amplicons were electrophoresed on 1% (w/v) agarose gels, stained with ethidium bromide, and visualized under UV light (Bio-Rad electrophoresis system). PCR products were purified (Zymo DNA Clean & Concentrator Kit) to remove primer dimers and sequenced on an Illumina MiSeq platform (San Diego, CA, USA). The 16S/26S rRNA gene sequences of all isolates were compared to the NCBI GenBank database using BLAST (http://www.ncbi.nlm.nih.gov/BLAST). Phylogenetic trees were reconstructed via the Neighbor-Joining method in MEGA v.11, incorporating sequences from the top five BLAST matches. Bootstrap analysis (1,000 replicates) validated node support. 

Whole-genome sequencing was performed on selected isolates exhibiting phenotypic traits of interest, WGS trimming, filtering, assembly, annotation, and functional analysis were carried out as described in Akinyemi et al. (2024a). Briefly, genomic libraries were prepared using the Illumina TruSeq Nano DNA Library Prep Kit and sequenced via paired-end (2 × 150 bp) Illumina NovaSeq 6000 (Novogene Bioinformatics Technology Co. Ltd., China), with library quality verified using a Qubit 2.0 Fluorometer (Thermo Scientific, USA). However, only the 16S rRNA gene sequences extracted from the assembled WGS data using extractseq software version 5.0.0 were utilized for phylogenetic and taxonomic analyses in this study (Rice et al. 2000). All sequences and assembled genomes were deposited in the NCBI database under accession numbers listed in the data availability section.

### Statistical analysis and visualisation tools

Biostatistical analysis and visualisation were conducted using R statistical software version 4.2.1, primarily with the Phyloseq package and its dependencies, unless otherwise specified. The descriptive statistics, including counts, mean, median, percentage, and range, were employed to describe the basic features of the sequences. Kruskal-Wallis analysis of variance was employed to evaluate differences in microbial richness (i.e., Chao1) and diversity (i.e., Shannon and Simpson) indices between goat breeds and farm management system. Differences among groups were determined using Dunn’s multiple comparison post-hoc test at alpha levels of 0.001, 0.01, and 0.05 presented as annotated boxplots. The Analysis of Group Similarities statistics and the Bray-Curtis distance method were used to assess the overall variance in microbial composition of the milk samples based on goat breed and goat farming system, and the results were visualised as Principal Component Ordinate Analysis (PCoA) plots. To elucidate the variation observed in the milk microbiota, variance partitioning was performed on the “variancePartition” and “vegan” r package using a combination of intrinsic and extrinsic variables. The intrinsic variables included metadata on the age, lactation period, and breed of each goat, which were collected from the respective dairy farms. Extrinsic variables consisted of climatic factors such as temperature, rainfall, humidity, and transpiration, which were sourced from the Climate Data Store (https://cds.climate.copernicus.eu/).

The relative abundance of phyla in each group was depicted as stacked percentage bar plots, while the genera was presented in percentages as pie charts and heatmaps. Linear discriminant analysis (LDA) Effect Size (LEfSe) was carried out using the “lefser” package at alpha level of 0.1. Co-occurrence of bacterial and yeast taxa was determined using the “NetCoMi” package and the Spearman correlation method, with a filter threshold of 0.01. Undirected network modularity was calculated using the igraph cluster fast greedy function. The resulting network and network attributes were imported and visualised on Gephi software version 0.10.1, except for taxa roles, which was visualised using the R package “ggplot2”. The functional traits of the genus grouped in each module on the network were calculated as percentages by querying against the FAPROTAX database without considering the abundance of the genus within each module.

The multivariate analysis of variance and Bonferroni’s post-hoc test was employed to determine differences in mycotoxin concentrations between ranched and nomadic goat milk. Canonical correlation analysis was performed and visualized using OriginPro 2024 to determine the association between mycotoxin concentrations and the top abundant bacterial and yeast genera at an alpha level of 0.05.

All culture dependent functional and safety experiments were conducted in triplicate, with results expressed as mean ± standard deviation (SD) for quantitative assays (e.g., survival percentages, auto-aggregation, hydrophobicity) or as categorical outcomes (presence/absence of enzymatic or fermentation activity).

## Result

### Sequence abundance

Following quality filtering and trimming, the 16S rRNA and 26S rRNA gene sequencing yielded 2,254,573 high-quality ASVs, comprising 1,030,531 bacterial and 1,224,042 yeast reads. Bacterial read counts averaged 39,635.81 per sample (median: 39,098; range: 15,835–74,826), while yeast reads averaged 47,078.54 per sample (median: 45,713.5; range: 16,380–118,273) (Fig. S1). Rarefaction analysis indicated that sequencing depth was sufficient for both bacterial and yeast communities, as all samples approached saturation (Fig. S2). The resulting ASVs were classified into 742 bacterial and 960 yeast database-featured taxa names. All annotated bacterial reads were classified at the phylum and class levels, 99.997% at the order level, 99.983% at the family level, and 97.62% at the genus level. For yeast reads, 64.8%, 45.68%, 43.18%, 34.86% and 34.56% were classified at the phylum, class, order, family, and genus levels, respectively. Only taxa with an average relative abundance of over 1% were considered dominant.

### Alpha diversity analysis

**Fig. 2:**
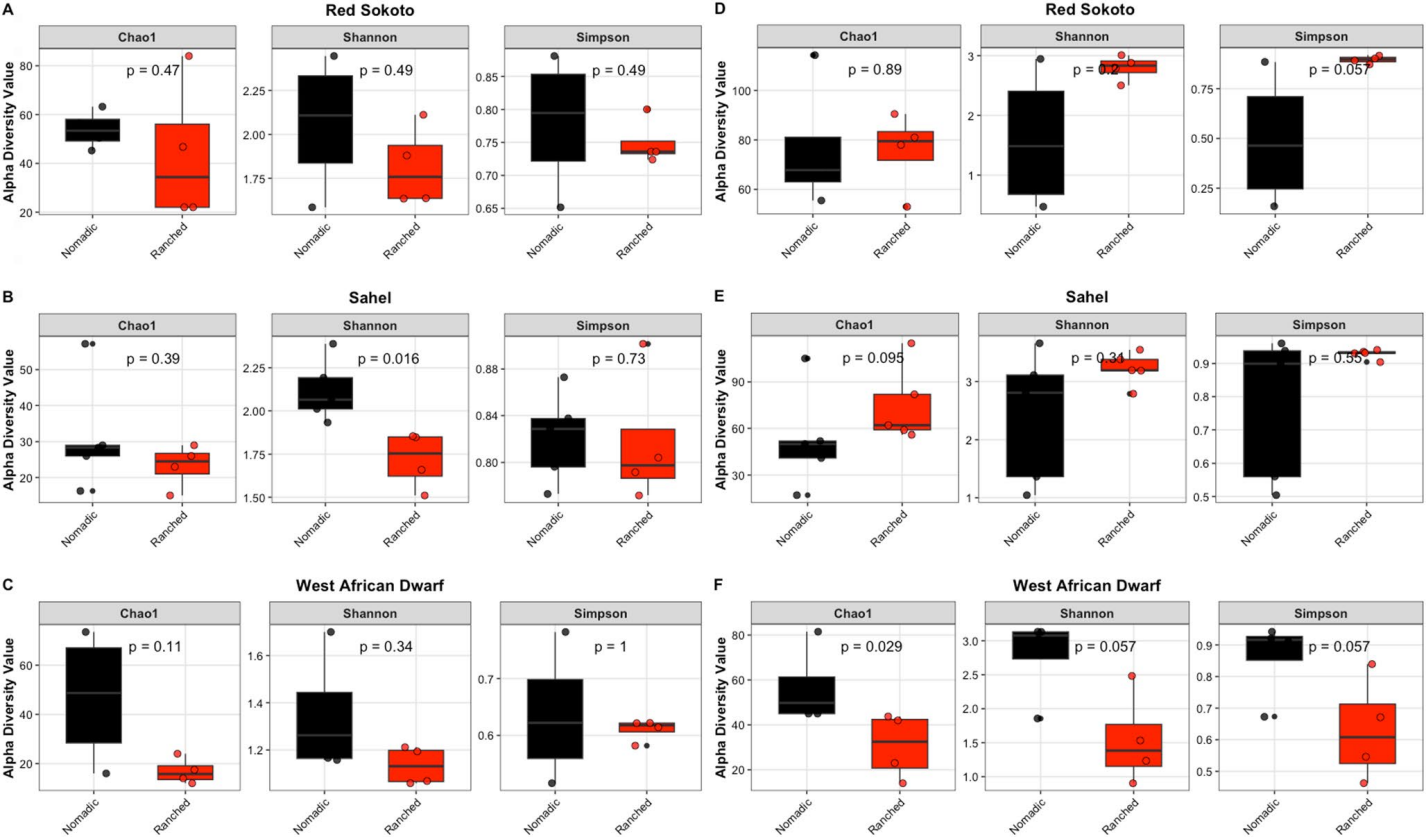
Alpha diversity box plots for **A**, **B**, **C**) bacterial and **D**, **E**, **F**) yeast communities in milk samples compared based on the goat farming system of **A**, **D**) Red Sokoto, (**B**, **E**) Sahel and **C**, **F**) West African Dwarf breed. Difference between the samples, compared using the Mann-Whitney U t-test is represented by “ns” for not significant and an Asterisks indicate statistical significance: (***) *p* < 0.001, (**) *p* < 0.01, (*) *p* < 0.05

Microbial alpha-diversity was estimated using the ASV richness (Chao1), Shannon–Wiener and Gini-Simpson diversity indices; with results visualized as boxplots (Fig. 2 and S3). Overall, the Chao1 abundance estimator revealed no significant variations in the richness of bacterial and yeast communities in the milk samples across the different goat breeds (ANOVA: *p >* 0.05), except for a significantly higher abundance of yeast in milk from the Red Sokoto breed (Chao1 mean: 79) compared to the West African Dwarf breed (Chao1 mean: 44) (ANOVA: *p* < 0.05, Fig. S3B). When stratified by farming system (nomadic vs. ranched), the mean Chao1 index showed that the milk of the nomadic West African Dwarf, Red Sokoto and Sahel had higher bacterial (Fig. 2A, B, C) ASVs compared to the ranched counterparts, while the inverse was observed in the yeast community except in West African Dwarf goat milk samples, where nomadic goats had significantly higher yeast richness (t-test: *p* < 0.05) (Fig. 2F).

Both the Shannon–Wiener and Gini-Simpson indices revealed significantly higher bacterial community diversity in the Red Sokoto, and Sahel breeds compared to the West African Dwarf breed (*p* < 0.05, Fig. S3A). Additionally, the Gini-Simpson index indicated a statistically significant difference in the dominance of yeast ASVs, with milk from the Sahel breed showing greater dominance of prevalent yeast taxa compared to milk from the Red Sokoto breed (ANOVA: *p* < 0.05, Fig. S3B). Similar to the Chao1 indices, each breed when stratified by farming system showed that milk of the nomadic goats had higher bacterial diversity compared to their ranched counterparts, while the inverse was observed in the yeast community except in milk from the West African Dwarf goats (Fig. 2). However, only bacterial richness in nomadic Sahel breed milk indicated statistical significance (t-test: *p* < 0.05).

### Beta diversity analysis

**Fig. 3:**
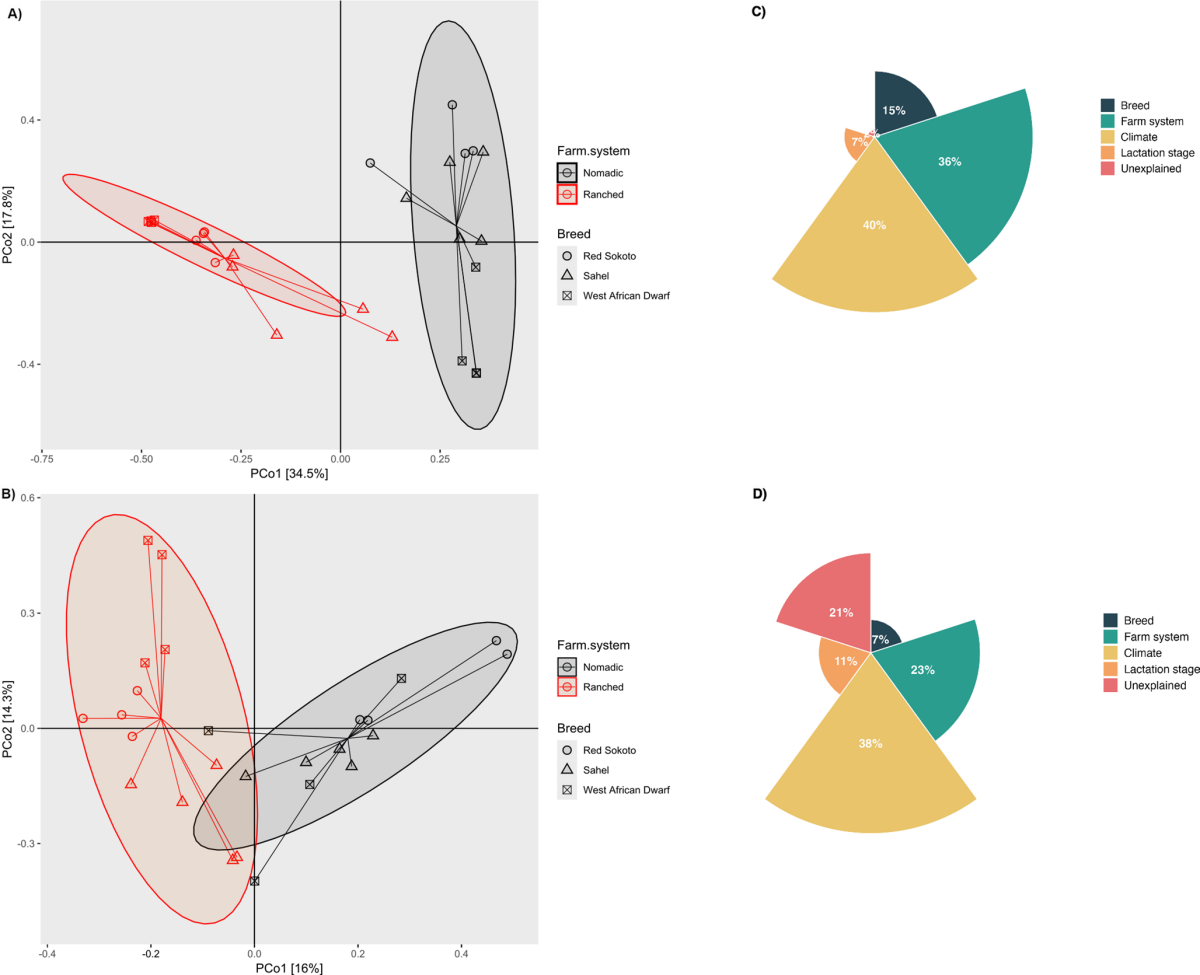
PCoA plot illustrating **A**) bacterial and **B**) yeast communities in goat milk samples. In the scatter plot, colour represents the breed, shape indicates the farming method. Radial plot illustrating **C**) bacterial and **D**) yeast variance partitioning

Beta diversity, assessed via Bray–Curtis dissimilarity and visualized using principal PCoA, demonstrated distinct clustering patterns for bacterial and yeast communities (Fig. 3A-B). The principal component analysis accounted for 51.1% and 30.3% of the total variance in bacterial and yeast communities, respectively, emphasising clustering patterns linked to breed-specific and farming practice-related factors (Fig. 3). To identify other drivers of observed variance among milk samples, a variance partitioning analysis was performed, incorporating extrinsic (climate, farming system) and intrinsic (breed, lactation stage) variables (Fig. 3C-D). Extrinsic factors accounted for 36% and 40% of the variance in bacterial communities (Fig. 3C), and 23% and 38% in yeast communities (Fig. 3D), respectively. In contrast, intrinsic factors explained 15% (breed) and 7% (lactation stage) of bacterial community variance (Fig. 3C), compared to 7% (breed) and 11% (lactation stage) for yeast communities (Fig. 3D). A residual 2% (bacterial) and 21% (yeast) of the variance remained unexplained by the factors analysed.

A total of 41 bacterial and 50 yeast ASVs were shared across the three breeds, constituting 86% and 83.5% of total sequence reads, respectively (Fig. S4i). Unique ASVs were identified for each breed: Red Sokoto goats had 163 bacterial and 315 yeast ASVs, Sahel goats had 73 bacterial and 324 yeast ASVs, and West African Dwarf goats had 86 bacterial and 195 yeast ASVs (Fig. S4). Consistent with alpha diversity trends, Red Sokoto and Sahel breeds shared more bacterial (26 ASVs) and yeast (45 ASVs) taxa compared to West African Dwarf, which shared fewer than 12 bacterial and 18 yeast ASVs with the other breeds (Fig. S4). These shared as well as unique ASVs explain the overlapping clustering of bacterial (Fig. 3A) and yeast (Fig. 3B) communities between Red Sokoto and Sahel breeds, contrasting with the distinct clustering observed for West African Dwarf. Notable differences in microbial communities were observed between nomadic and ranched goats within each breed, as visualized by PCoA clustering (Fig. 3A-B). Bacterial and yeast communities from similar farming systems exhibited closer clustering, with 61–89% of total ASVs shared between nomadic and ranched goats. Nomadic goats harboured 7–32% unique bacterial ASVs, exceeding those in ranched counterparts across all breeds. A comparable trend was observed for yeast ASVs, except in the Sahel breed, where ranched goats displayed higher unique yeast ASVs (*n* = 233) than nomadic individuals (*n* = 153) (Fig. S4).

### Community composition

**Fig. 4:**
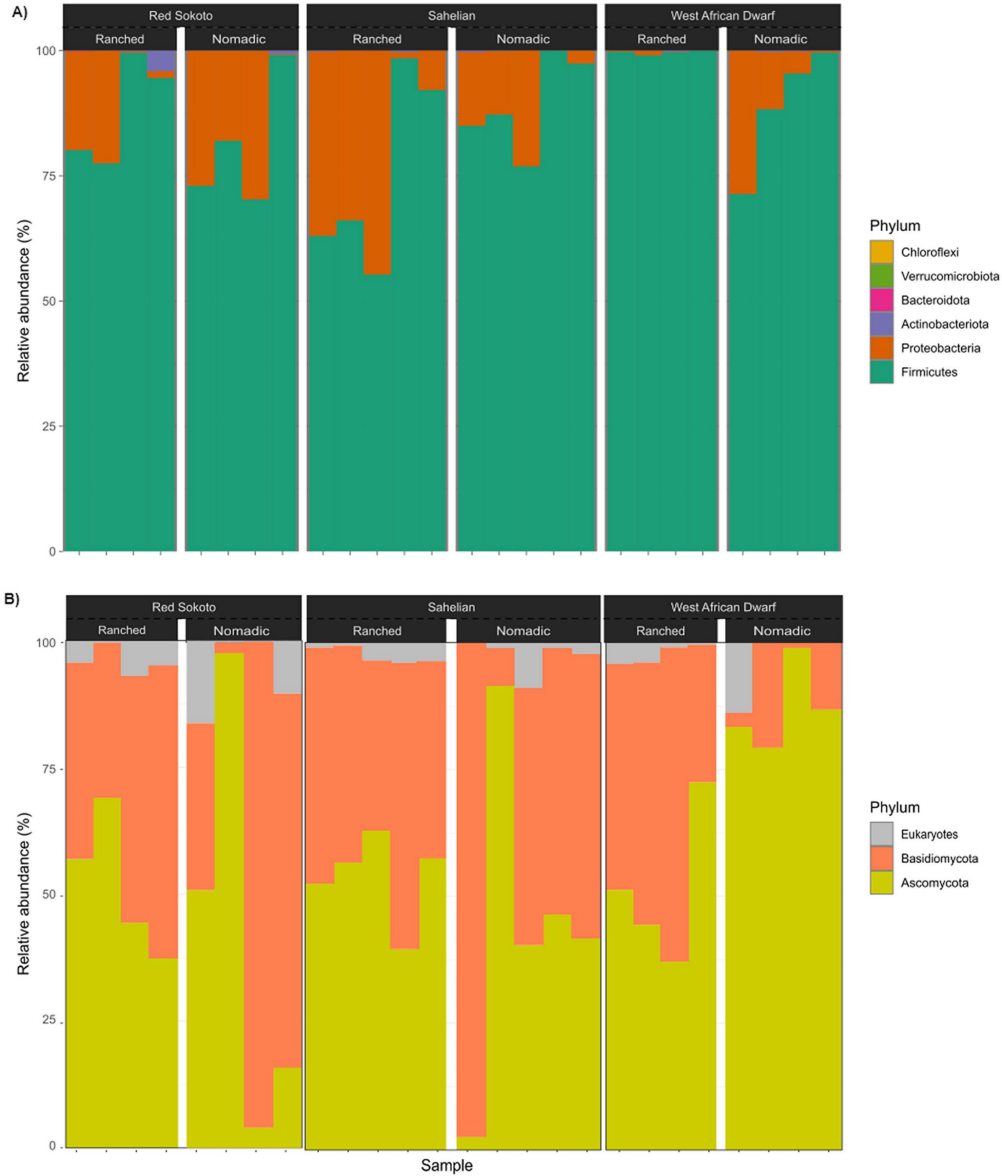
Relative abundance of **A**) bacterial and **B**) yeast phyla across the milk samples of Red Sokoto, Sahel, and West African Dwarf goat breeds from ranched and nomadic farming systems. The percentage of sequences is plotted on Y-axis

A comprehensive taxonomic analysis of milk samples revealed six bacterial and two fungal phyla (Fig. 4). Among bacterial phyla, Firmicutes (also known as Bacillota) dominated with a relative abundance of 86.5%, followed by Proteobacteria (also known as Pseudomonadota) (13.2%) and Actinobacteriota (0.3%), collectively accounting for over 99% of all bacterial sequences (Fig. 4A). The remaining bacterial phyla Bacteroidota, Verrucomicrobiota, and Chloroflexi (also known as Chloroflexota) were detected at trace levels (< 0.1% abundance). Fungal communities were primarily structured by Ascomycota (55%) and Basidiomycota (44%), with the latter representing a significant but minority component of the yeast microbiota (Fig. 4B).

**Fig. 5:**
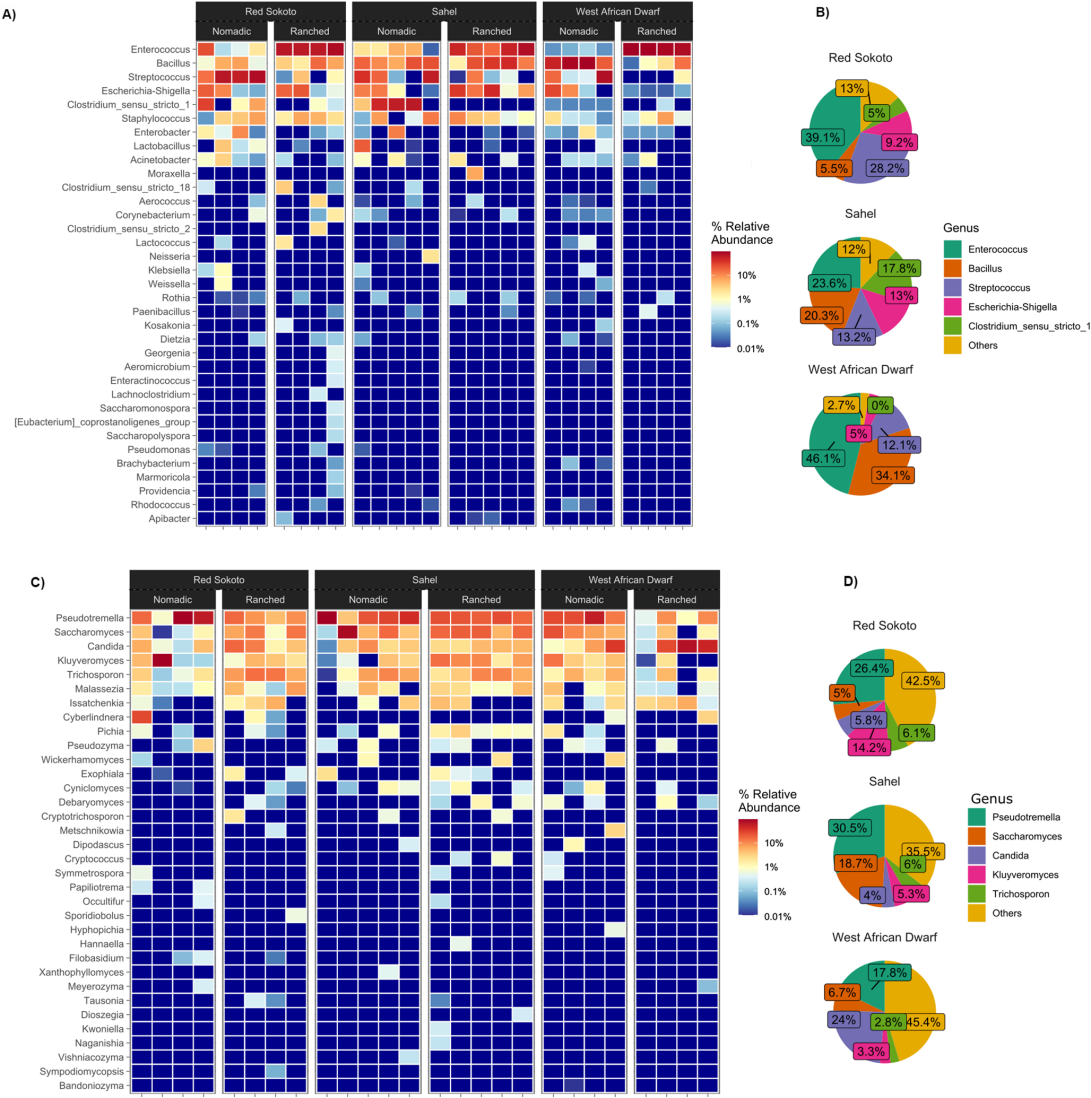
Relative abundance of **A**) bacterial and **C**) yeast genera across the milk samples of Red Sokoto, Sahel, and West African Dwarf goat breeds from ranched and nomadic farming systems. The percentage abundance of the top genera is plotted as pie charts in figure **B** and **D**, respectively

**Fig. 6:**
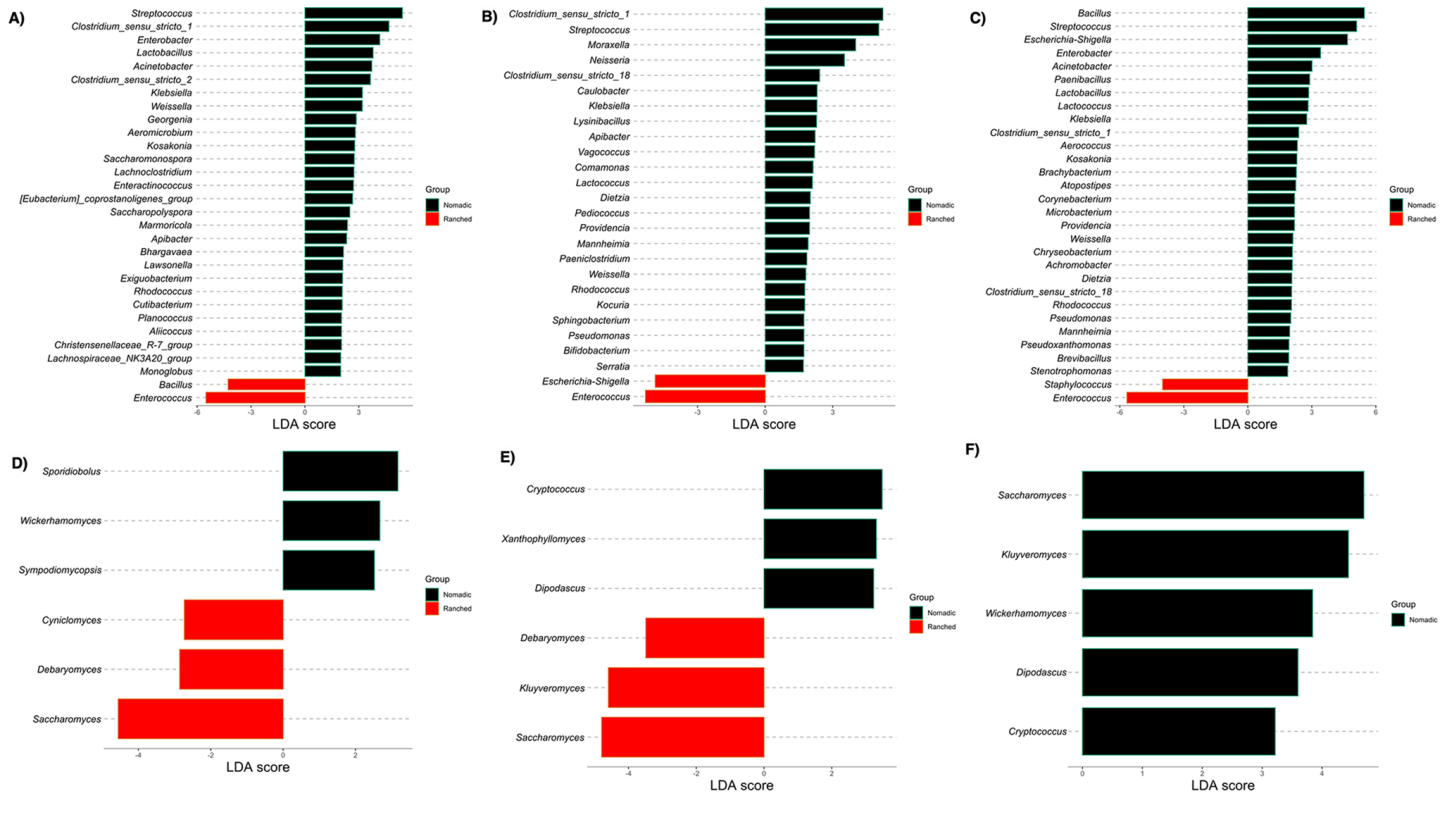
Effect of size measurements (LEfSe) of (**A**, **B**, **C**) bacteria and (**D**, **E**, **F**) yeast at genus across the milk samples of (**A**, **D**) Red Sokoto, (**B**, **E**) Sahel, and (**C**, **F**) West African Dwarf goat breeds from ranched and nomadic farming system

At the genus level, bacterial communities exhibited a clear dominance hierarchy. The most abundant genera included *Enterococcus* (35.3%), *Bacillus* (20.0%), *Streptococcus* (17.5%), *Escherichia-Shigella* (9.4%), and *Clostridium sensu stricto 1* (8.4%) (Fig. 5A-B). These five genera collectively represented 90.6% of all bacterial sequences (Fig. 5B), reflecting a highly structured core microbiota. Fungal communities, conversely, were dominated by *Pseudotremella* (38%), *Saccharomyces* (35%), *Candida* (12%), *Kluyveromyces* (4%), and *Trichosporon* (4%), which together accounted for 93% of fungal sequences (Fig. 5C-D). Distinct patterns emerged in bacterial community composition based on farming practices. In nomadic systems, differentially abundant bacterial genera included *Streptococcus*, *Clostridium*, and *Lactobacillus*, with Linear Discriminant Analysis (LDA) scores ranging from 2 to 4 (Fig. 6A-C). Additionally, there was a relatively higher abundance of genera with known species of concern, such as *Streptococcus*, *Acinetobacter*, *Klebsiella*, and *Pseudomonas* (Figs. 5A and 6A-C). These genera are often associated with environmental exposure and traditional grazing practices. In contrast, ranched systems exhibited a shift toward *Enterococcus* as the dominant genus, with LDA scores between 2 and 3 (Fig. 6A-C). For instance, in ranched Red Sokoto goats, *Enterococcus* constituted 39.1% of bacterial sequences, compared to 23.6% in Sahel and 46.1% in West African Dwarf breeds (Fig. 5B). Notably, this pattern was consistent across all three breeds, suggesting that farming system plays a significant role in shaping bacterial community structure, potentially overriding breed-specific factors.

Similar to bacterial communities, yeast profiles varied by farming system but were more consistent across breeds (Fig. 5C). In nomadic systems, *Saccharomyces* and *Cryptococcus* were the most prevalent yeast genera, with LDA scores ranging from 2 to 4 (Fig. 6D-F). In ranched systems, *Saccharomyces* remained dominant, but *Debaryomyces* replaced *Cryptococcus* as a core genus, with LDA scores between 2 and 3 (Fig. 6D-F). Similar to bacterial communities, yeast profiles were consistent across breeds within the same farming system. The consistency of these patterns across all three breeds suggests that farming system is a dominant ecological driver of milk microbiota. However, the relatively modest LDA scores (2–4) indicate that while these genera are statistically significant, their dominance is not absolute, and other factors likely contribute to microbial community composition.

### Co-occurrence network and predictive functionality of bacteria and yeast strains

**Fig. 7:**
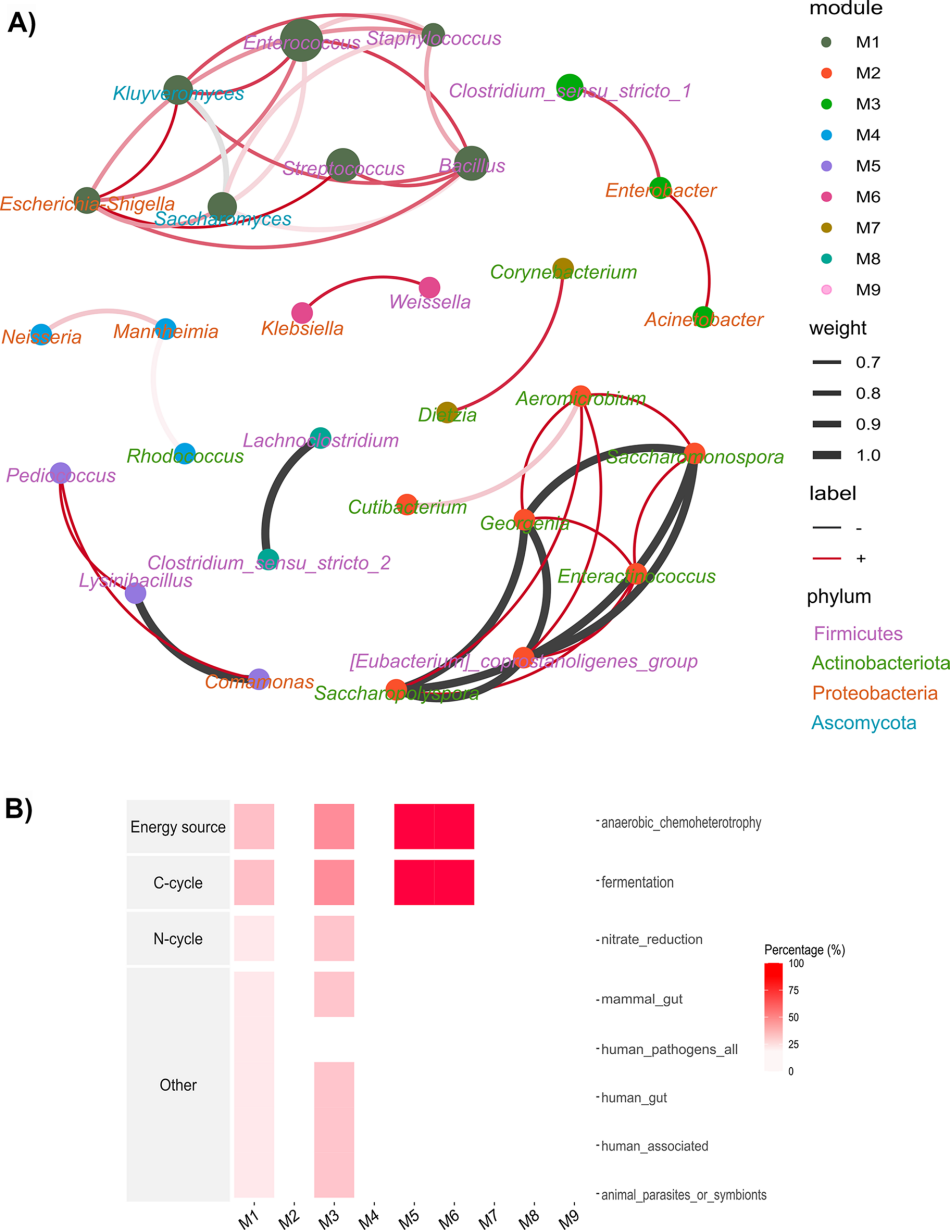
Co-occurrence network of **A**) bacteria and yeast in milk samples of Red Sokoto, Sahel, and West African Dwarf goat breeds and **B**) predictive functionality of genera within each module. Nodes of the network are coloured by phylum with size of the node corresponds to relative abundance of the genera

To elucidate interactions between bacterial and yeast communities in milk, a co-occurrence network was constructed (Fig. 7) and the topological properties of the network analysed (Table S1). The network comprised 29 genus-level nodes connected by 43 edges, with a modularity score of 0.697 and a Leiden algorithm quality score of 0.98, indicating robust community structure (Fig. 7, Table S1). Nodes were clustered into nine distinct modules, reflecting groups of taxa with shared ecological roles or environmental dependencies. The majority of edges (35/43, 81.3%) represented positive correlations, while 8/43 (18%) indicated negative interactions. Nodes were taxonomically assigned to four dominant phyla: Proteobacteria (31.63% of nodes), Firmicutes (23.47%), Actinobacteriota (10.2%), and Ascomycota (5.1%). All nodes were classified as peripheral based on Zi (within-module connectivity) and Pi (between-module connectivity) thresholds, suggesting limited influence on overall network stability (Fig. S5). Positive correlations were predominantly observed within and between genera belonging to Ascomycota, Firmicutes, and Proteobacteria, reflecting potential synergistic relationships. Key modules included Module 1, which contained genera such as *Bacillus*, *Escherichia-Shigella*, *Enterococcus*, *Kluyveromyces*, *Saccharomyces*, *Staphylococcus*, and *Streptococcus*, and Module 3, which included *Acinetobacter*,* Enterobacter*, and *Clostridium*. Functional predictions revealed that 25% of genera in module 1 were associated with nitrate reduction and 20% were predicted to be human pathogens, while 45% of genera in module 3 were linked to nitrate reduction and 32% were predicted to function as animal or human symbionts/parasites (Fig. 7). All genera in module 5, which comprised of *Lysinibacillus*, *Pediococcus* and *Klebsiella*, as well as module 6 that contained *Weissella*, *Corynebacterium* and *Dietzia* were predicted to be capable of utilizing organic molecules as primary carbon and energy sources via anaerobic fermentation (Fig. 7).

### In vitro characterisation of safety and functional properties of bacteria and yeast strains

**Fig. 8:**
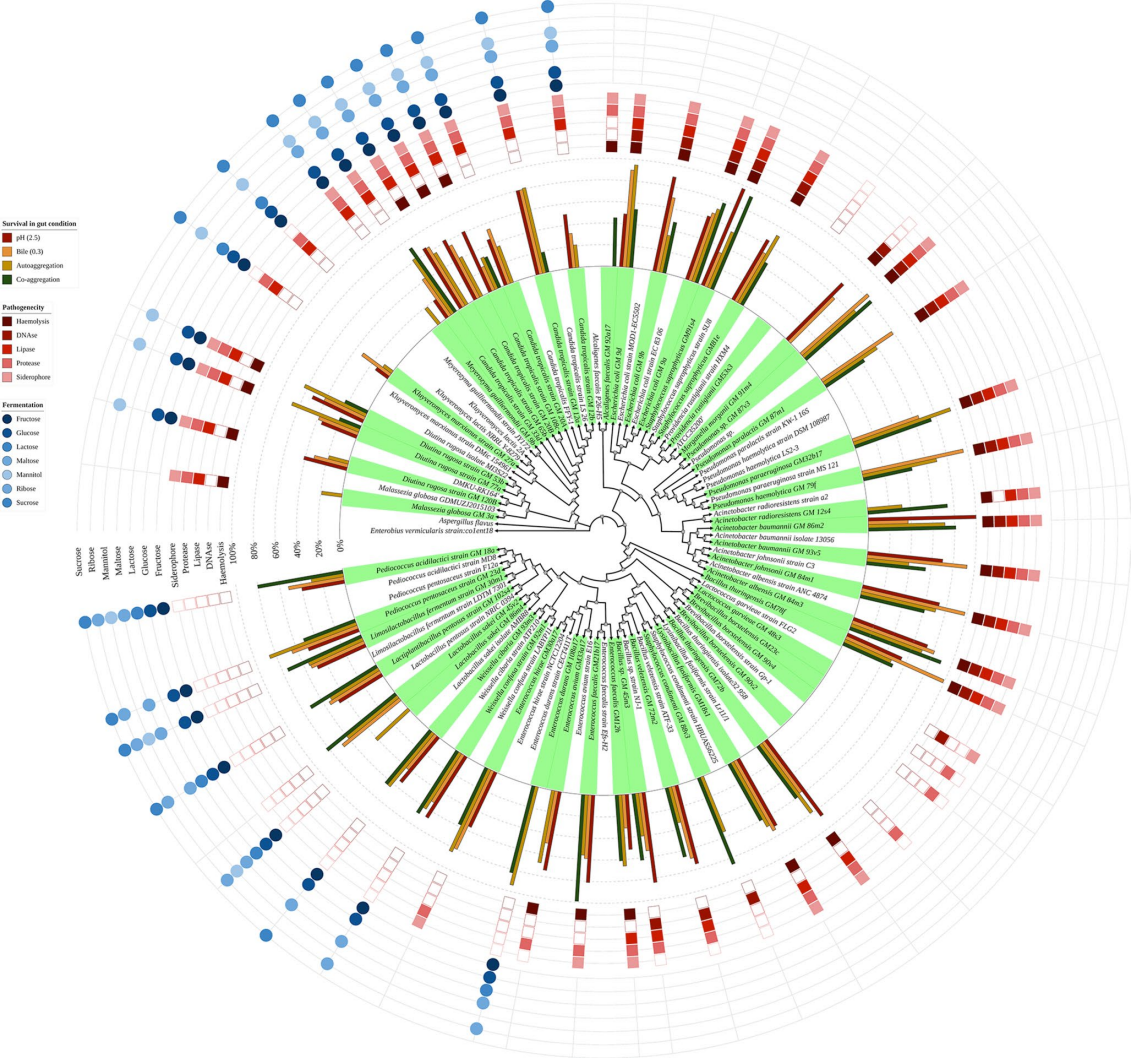
Phylogenetic relationships and functional characteristics of bacterial isolates. Starting from the inner ring: (ring 1) Maximum-likelihood phylogenetic tree constructed from 16S rRNA gene sequences, highlighting the evolutionary placement of studied strains (clades shaded in green) relative to reference strains retrieved from NCBI GenBank (unshaded clades). (ring 2) Survival rates of isolates under simulated human gastrointestinal tract (GIT) conditions, including acidic pH (2.5) and bile salts (0.3%), alongside GIT colonization efficiency (auto-aggregation and hydrophobicity). (ring 3) Pathogenicity-associated phenotypes, including haemolytic activity, enzymatic profiles (DNAse, lipase, protease), and siderophore production. (ring 4) Carbohydrate fermentation profiles of non-pathogenic isolates, indicating substrate utilization (fructose, glucose, lactose, maltose, mannitol, ribose, sucrose)

The in vitro phenotypic confirmation of the predicted functional and safety properties of 41 bacterial and 12 yeast strains are presented in Fig. 8. The isolates, representing species of diverse bacterial genera such as *Acinetobacter*,* Alcaligenes*,* Bacillus*, *Brevibacillus*, *Escherichia*,* Lactobacillus*,* Lactococcus*,* Limosilactobacillus*, *Lactiplantibacillus*, *Lactobacillus*, *Lysinibacillus*, *Morganella*, *Pediococcus*, *Pseudomonas*, *Providencia*, *Staphylococcus*,* Weissella*, and fungal genera including *Candida*, *Diutina*, *Kluyveromyces*, *Malassezia* and *Meyerozyma*, were evaluated across three primary categories: (1) survival under gastrointestinal stress conditions, (2) virulence-associated phenotypes, and (3) carbohydrate fermentation profiles.

Bacterial isolates exhibited variability in stress tolerance and functional traits. Species within the Lactobacillaceae family, including *Limosilactobacillus fermentum* (strain 30m1), *Lactiplantibacillus pentosus* (102s4), *Weissella confusa* (92m1), *Weissella cibaria* (93m3), and *Pediococcus* spp. (23d and 18a), demonstrated robust survival under acidic conditions (pH 2.5 survival: 49–67%) and bile salt tolerance (0.3% survival: 41–65%). These strains also displayed strong auto-aggregation (30–86%) and hydrophobicity (51–80%), properties linked to probiotic potential, such as adhesion to intestinal epithelia. Some strains from these species were tested for probiotic properties. The full probiotic potential of *L. fermentum* (isolate 30m1), *Lp. pentosus* (102s4), *W. confusa* (92m1), *W. cibaria* (93m3), *Pd. pentosaceus* (23d), and *Pd. acidilactici* (18a) was analyzed in detail in a previous study (Akinyemi et al. 2022b). Moreover, all Lactobacillaceae isolates were non-pathogenic, lacked haemolytic activity (gamma-haemolysis), and did not produce DNAse, lipase, or protease. In contrast, pathogenic and opportunistic-pathogenic bacteria, including *Escherichia coli* (strains 9a, 9d and 9b), *Acinetobacter* spp. (84m1, 84m3, 86m2 and 93v5), *Staphylococcus saprophyticus* (81e and 91s4), and *Bacillus thuringiensis* (72b and 78f), exhibited high stress tolerance with pH and bile survival rate ranging between 30 and 100% (Fig. 8). These strains also tested positive for protease, lipase, siderophore production, and haemolytic activity (alpha-, or beta-haemolysis). For example, *E. coli* strains displayed alpha- or beta-hemolysis and siderophore production, while *B. thuringiensis* (72b and 78f) showed alpha-hemolysis. Opportunistic pathogens such as *Alcaligenes faecalis* (92a17), *Enterococcus hirae* (30a17) and *Pseudomonas paraeruginosa* (32b17) exhibited lack of tolerance to pH and bile conditions of human GIT, however, both strains were haemolytic, iron and protein scavengers while *P. paraeruginosa* (32b17) also produced DNAse and lipase enzyme.

There were also non-pathogenic bacterial isolates outside the Lactobacillaceae family which exhibited limited tolerance to simulated gastrointestinal stress conditions. For instance, *Staphylococcus condimenti* (strain 88v3), *Brevibacillus borstelensis* (90v4, 90v2 and 23c), *Enterococcus durans* (108a17), *Providencia rustigianii* (53s3) and *Lactococcus garvieae* (48s3) demonstrated 0% survival at pH 2.5 and in the presence of 0.3% bile salts, indicating poor resilience to acidic gastric environments and intestinal bile. Virulence factor analysis revealed that these strains also generally lacked overt pathogenic traits. *Staphylococcus condimenti* (strain 88v3) and *E. durans* (108a17) were non-haemolytic (gamma-haemolysis) and negative for DNAse, lipase, and protease production, aligning with their classification as non-pathogens.

Non-pathogenic isolates, including those from the Lactobacillaceae family, varied in their ability to ferment different sugars. All non-pathogenic isolates could ferment glucose, fructose, and maltose. Except for *W. cibaria*, all other isolates fermented ribose. *Pediococcus acidilactici* GM18a stood out as the only strain able to ferment all seven tested sugars, while *Weissella* species showed the weakest performance, fermenting only four sugars each.

Yeast isolates, including *Candida tropicalis*, *Diutina rugosa* and *Kluyveromyces marxianus*, showed variable responses to stress and functional assays. *Candida tropicalis* strains (e.g., 12b, 108c and 20 A) displayed moderate pH 2.5 tolerance (45–80%) and bile resistance (24–80%), with hydrophobicity ranging from 19 to 72%. Auto-aggregation was inconsistent (10–81%), though strain 12 A exhibited 81% auto-aggregation. All *C. tropicalis* strains produced protease and siderophores, and most were positive for lipase, though their pathogenicity classification varied. *Kluyveromyces marxianus* (strain 27a) showed low stress tolerance (pH 2.5: 14%; bile: 32%) and lacked lipase activity, while *D. rugosa* (strains 53b and 77a) exhibited beta-hemolysis and moderate hydrophobicity (21–23%). *Kluyveromyces lactis* (strain 2a) and *Meyerozyma guilliermondii* (61 C) displayed limited enzymatic activity. Carbohydrate fermentation profiles further differentiated yeast strains. *Candida tropicalis* isolates fermented fructose, glucose, and maltose but not lactose, while *K. marxianus* (strain 27a) fermented fructose and glucose but not lactose or maltose. *Diutina rugosa* (strains 53b and 77a) showed limited fermentation capacity, failing to metabolize lactose, maltose, or mannitol.

### Mycotoxins in goat milk and predicted association with potential technological microbiota

In a prior study, we reported the presence and concentrations of several mycotoxins in raw milk samples detected by LC-MS/MS analysis (Akinyemi et al. 2022a). To explore the potential role of milk microbiota in modulating mycotoxin dynamics, the present study paired milk microbiota with mycotoxins detected in milk samples. This approach prioritized farming system as a variable due to its previously demonstrated dominance over breed in shaping microbial communities.

**Fig. 9:**
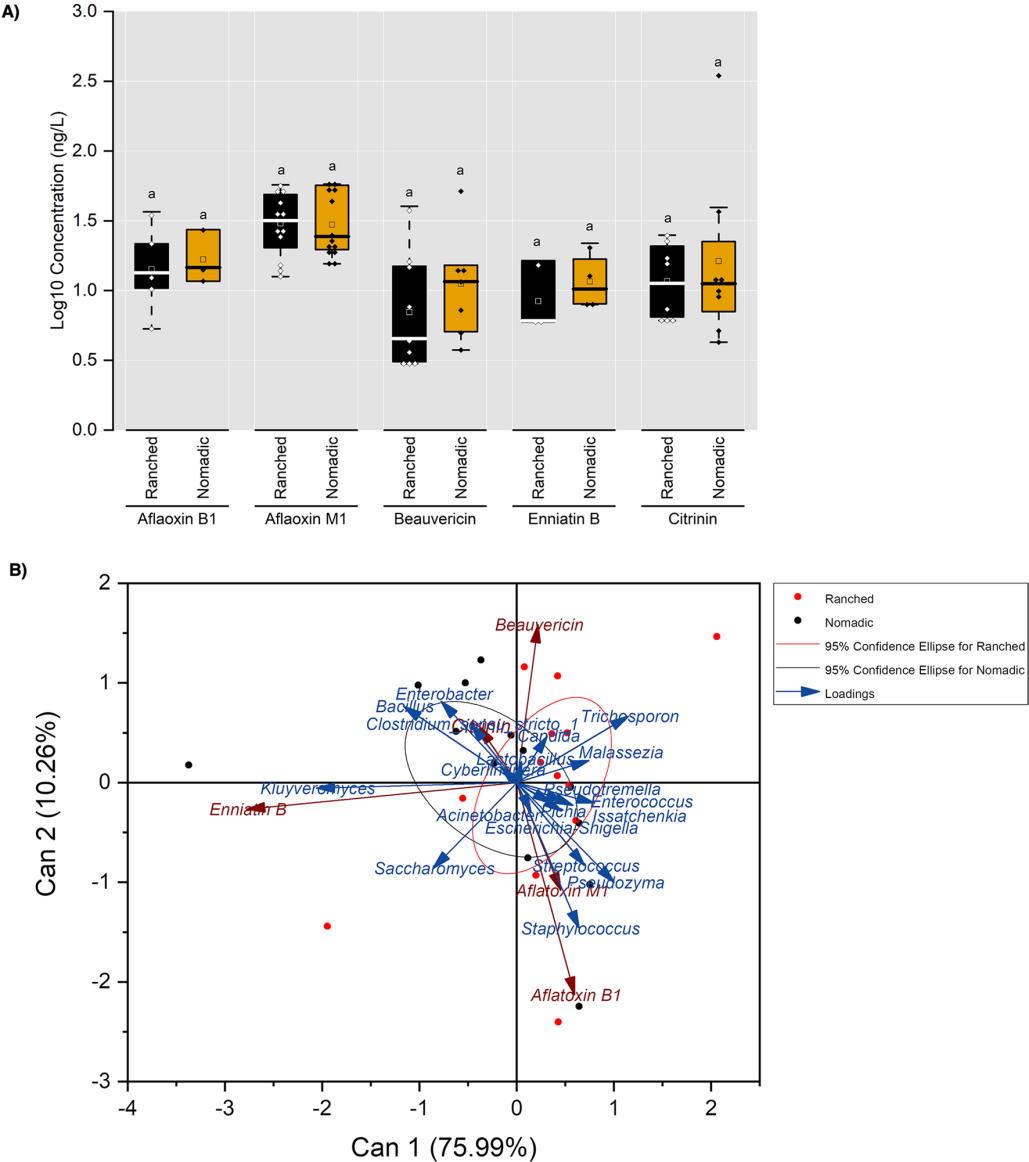
**A**) Variation in mycotoxin levels in milk samples of Red Sokoto, Sahel, and West African Dwarf goat breeds from ranched and nomadic farming systems. The letters above the boxes denote differences between the concentrations of each mycotoxin in ranched and nomadic goat milk at alpha level of 0.05. **B**) Loading plot and clusters of Canonical Correlation Analysis (CCA) of top dominant bacteria and yeast at the genus level and major mycotoxins in milk samples from Red Sokoto, Sahel, and West African Dwarf goat breeds

Seven mycotoxins, namely: aflatoxin B_1_ (AFB_1_), aflatoxin M_1_ (AFM_1_), aflatoxin P_1_, beauvericin (BEA), enniatin B (EnnB), citrinin, and ochratoxin A; were detected at concentrations ranging from 5.3 to 362 ng/L (Table S2). AFM_1_ was the most prevalent (96% of samples), though levels (12.5–57.9 ng/L) remained below the 500 ng/L regulatory threshold in Nigeria. Notably, AFB_1_, a potent carcinogen, was detected in milk from both farming systems. No significant differences (t-test: *p* > 0.05) in mycotoxin concentrations were observed between ranched and nomadic milk samples (Fig. 9).

Canonical correlation analysis (CCA) was employed to identify microbial genera associated with variations in mycotoxin concentrations. The first two CCA axes explained 86.25% of the variance (Axis 1: 75.99%; Axis 2: 10.26%), revealing correlations between specific taxa and mycotoxin profiles (Fig. 9B; Supplementary File 1). Lower AFB_1_ levels revealed low (0.09 < r^2^ < 0.25) to moderate correlations (0.25 < r^2^ < 0.49) with higher abundances of *Escherichia/Shigella*, *Streptococcus*, *Staphylococcus*, *Saccharomyces*, and *Pseudozyma*. A similar pattern of correlation was observed between AFM_1_ and *Enterobacter*,* Staphylococcus*,* Streptococcus*,* Pichia*, and *Pseudozyma*. *Bacillus* and *Malassezia* correlated with reduced BEA levels, while *Bacillus* and *Kluyveromyces* were linked to lower EnnB concentrations.

## Discussion

Microbial community composition and structure are important influencers of the overall health of animals and humans who consume animal products (Oikonomou et al. 2020). Factors such as animal’s diet, breed, lactation stage, antimicrobial use, feed management, geographic area, seasonality, environmental and housing conditions can impact the composition and diversity of the microbial community in ruminant milk (Oikonomou et al. 2020; Guo et al. 2024). This study demonstrates that farming systems, specifically ranching versus nomadic practices, exert a more significant influence on milk microbiota than breed-specific factors. As far as we know, this study is the first to: (a) apply next-generation sequencing techniques to investigate microbial taxa in these breeds; and (b) compare the influence of ranching and nomadic goat farming systems on the microbial communities in a sub-Saharan Africa context.

While alpha diversity indices indicated that Red Sokoto and Sahel goats, raised in the arid Sahel region of Sokoto, exhibited higher bacterial diversity compared to the West African Dwarf breed from the savanna (Kwara state), these differences likely reflect geographic and climatic variations rather than inherent breed characteristics. The extreme temperatures, exceeding average daily temperature of 45 °C during the sampling period in Sokoto state may select for stress-tolerant microbial taxa, whereas the milder savanna climate (average daily temperature: 30°) in Kwara State supports distinct ecological niches. This aligns with global studies linking climatic conditions to feed microbiota and subsequent milk microbial diversity (Parente et al. 2020).

Within the goat species, farming practices further modulated microbial profiles. Our findings indicates that nomadic goats harboured milk microbiota with higher richness and unique ASVs compared to ranched goats fed standardized diets. The higher diversity in nomadic goat breeds may be attributed to feed types, which is largely determined by the goat farming system employed. This disparity underscores the role of diet in introducing environmental microbes into milk (Hoving-Bolink et al. 2023). In this present study, ranched goats were typically fed a controlled and balanced diet that included pre-defined/specific types of feed, such as commercial pellets, legumes, pulse, oilseeds and sometimes supplements such as minerals and vitamins. Such controlled feeding ensures that the goats receive a consistent and nutritious diet, which impacts the composition of their milk and the associated microbial diversity. Goats in nomadic farming systems often forage for their food, which can include a variety of plants, shrubs, and even household food waste. Thus, the diet of nomadic goats is more diverse and variable, depending on the availability of natural feed in their environment. Obviously, the consumption of a wider range of feed types exposed these goats to a broader spectrum of microorganisms, which was reflected in the diversity of milk microbiota. These findings corroborate studies from China (Jing et al. 2021) and the Netherlands (Hoving-Bolink et al. 2023), where pasture-grazed goats exhibited richer microbiota than those on indoor diets.

While a diverse microbial population can have benefits such as enhancing the nutritional and probiotic value of goat milk, there is also the risk associated with pathogenic members of the community. At the phylum level, Firmicutes (86.5%) and Proteobacteria (13.2%) dominated bacterial communities, consistent with global dairy microbiota studies. For example, the dominant phyla found in this study were also reported in milk from Saanen and Guanzhong goats in China (Zhang et al. 2017). Similarly, McInnis et al. (2015) identified Firmicutes, Proteobacteria, Actinobacteria, and Bacteroidetes as the dominant phyla in Alpine, Toggenburg, Saanen, and LaMancha goats in the United States, while Kamilari et al. (2020) reported only Firmicutes and Proteobacteria in Shami goat milk from Cyprus. Also, both Ryu et al. (2021) and Lauková et al. (2022) reported the four phyla in goat milk from Korea and Slovakia, respectively.

It is no surprise that the phylum Ascomycota and Basidiomycota are present in milk since all known yeast types belong to these two taxonomic groups (Kurtzman 1994; Robert et al. 2013). Studies on fungal diversity in milk that employed internal transcribed spacer (ITS) based classifiers such as UNITE (Abarenkov et al. 2010), the hierarchical taxonomic classifier (HiTaC) (Miranda et al. 2024) or other approaches also identified yeasts within this group (Delavenne et al. 2011; Buehler et al. 2017). However, the prominence of Basidiomycota among yeast microbiota in milk remains understudied, highlighting a gap in fungal microbiota research.

Genus-level analysis revealed *Enterococcus* as the most abundant bacterial taxon, particularly in ranched goats. Previous studies reported *Enterococcus* as a dominant group in the milk of mammals, of which goat milk is no exception (McInnis et al. 2015; Lauková et al. 2020; Ryu et al. 2021). Although there are currently no reports on microbial diversity in goat feed in Nigeria, the higher prevalence of *Enterococcus* in ranched goats compared to nomadic goats may be attributed to the microbial composition of the feed of ranched goats. This group is commonly reported among microbes in goat feed components, such as cassava peel (Elijah et al. 2014; Anyogu et al. 2014) and maize (Fasusi et al. 2021). Nonetheless, additional studies are required to substantiate this hypothesis.

Conversely, nomadic goats exhibited higher abundances of *Bacillus*, *Streptococcus*, and *Clostridium* sensu stricto 1. These three groups are also well documented in studies which applied next generation sequencing to determine the microbial communities in milk (Callon et al. 2007; McInnis et al. 2015). Although little is known about the uncultured *Clostridium* sensu stricto 1, *Bacillus* and *Streptococcus* species are commonly found in the bovine gastrointestinal tract and faeces (Adetunji and Odetokun 2011; Kofoworola et al. 2014). Species of these genera can contaminate milk during the milking process and are frequently isolated from raw milk and milk products in Nigeria potentially leading to spoilage or food safety concerns when viable cells are present in large numbers (Ajayi et al. 2011; Amosun et al. 2018; Tolulope et al. 2024). Studies from various regions globally have reported similar results indicating that animal farming systems influence the variability in the milk microbiome. For instance, research on goat milk microbiota in China (Jing et al. 2021) and the Netherlands (Hoving-Bolink et al. 2023) demonstrated that goats grazing on pasture exhibited a greater diversity of microbial communities compared to those fed indoors on a controlled diet. The study concluded that the natural grazing environment introduced a broader range of microorganisms into the milk, aligning with the findings from the present study. A French study also reported that free grazing goats on natural pastures produced milk with a more diverse microbial profile, including a higher prevalence of beneficial lactic acid bacteria, in comparison to goats fed on a concentrated diet (Tormo et al. 2015).

The abundance of yeast genera underscores the complex microbiota typically present in dairy products. However, the existing data on the diversity of yeasts in dairy products is not as comprehensively documented as that for bacteria. *Pseudotremella*, the most dominant genera identified in this study, is an anamorphic basidiomycetous yeast not previously linked to milk (Liu et al. 2015a). Globally, four species of *Pseudotremella* are recognized, i.e., *P. allantoinivorans*,* P. lacticolor*,* P. moriformis*, and *P. nivalis*, two of which are only known in their yeast forms (Chen 1998; Liu et al. 2015a, b). This genus is more commonly found in environments such as soil, plants, and decaying wood, where they play roles in decomposition and nutrient cycling (Chen 1998). While it is theoretically possible for fungal spores to contaminate milk, especially if the milking equipment or environment is not properly sanitized, *Pseudotremella* specifically is not commonly reported as a contaminant in milk or milk products. On one hand, among the top abundant yeasts commonly associated with dairy environments, are the technologically important *Saccharomyces* and *Kluyveromyces* group (Delavenne et al. 2011; Quintana et al. 2020; Qvirist et al. 2016). These genera of yeast are frequently isolated from raw milk in Nigeria, playing important roles in food biotechnology and production of traditional cheese and yogurt (Jimoh et al. 2012; Onwuhafua et al. 2018). On the other hand, the presence and dominance of genera such as *Candida* and *Trichosporon* considered potentially pathogenic yeast may pose potential health concerns (Caetano et al. 2023). Although *Candida* is a part of the normal human microbiota, both genera, often reported in bovine milk, have been well documented as opportunistic pathogens (Sule & Kumurya 2019; Caetano et al. 2023; Oluranti et al. 2023).

Several other genera of concern were present in the investigated milk samples, though with lower prevalence. Differential abundance analysis (LefSe) revealed higher prevalence of bacterial and yeast genera such as *Escherichia*,* Clostridium*,* Corynebacterium*,* Neisseria*,* Klebsiella*,* Pseudomonas*,* Serratia*,* Staphylococcus*, and *Cryptococcus* in milk from nomadic goats. The higher prevalence of these microbial genera, many of which have known pathogenic species, is unsurprising given the diet of nomadic goats. For example, in vitro screening of isolates recovered from the milk confirmed the pathogenicity of strains of *B. thuringiensis*, *E. coli*, *S. saprophyticus*, and *A. baumannii* among others. These taxa exhibited virulence traits such as haemolysis, protease production, and siderophore activity, which enhance their ability to colonize hosts and evade immune responses. For instance, *Acinetobacter* spp. showed 100% survival at pH 2.5, indicating potential resilience in gastrointestinal environments. It is evident that the nomadic goat farming practice results in genera with well-established opportunistic human pathogens in milk. The occurrence and pathogenicity of *Acinetobacter*,* Escherichia*,* Klebsiella*,* Staphylococcus* and *Pseudomonas* species among others in bovine milk consumed in Nigeria has been well established (Ajayi et al. 2011; Amosun et al. 2012; Edward and Inya 2013; Christianah and Opadoyin 2016; Oladipo et al. 2016; Akinyemi 2024a). The finding in this study highlights a need to redefine goat farming practices in Nigeria, particularly the nomadic farming system.

Despite safety concerns, this study identified microbial strains with biotechnological promise. Lactic acid bacteria (LAB) such as *L. fermentum* (30m1) and *W. cibaria* (93m3) exhibited high auto-aggregation (95%) and hydrophobicity (84%), traits linked to probiotic adhesion and pathogen inhibition (Akinyemi et al. 2022b, 2024b). Similarly, *Pd. acidilactici* (18a) along with yeasts like *K. marxianus* (27 A), *K. lactis* (2a) and *M. guilliermondii* demonstrated broad carbohydrate fermentation capabilities, suggesting potential as a starter culture. Strains of similar species have been reported in previous studies and are known for their technological importance (Banwo et al. 2024; Shruthi et al. 2024; Remini et al. 2024). The co-occurrence of diverse microbial genera in milk presents an opportunity to harness them in the selection of probiotics, starter, and adjunct cultures in the production of traditionally fermented foods.

Mycotoxin contamination of dairy products represents a significant public health and economic concern in Nigeria, driven by the widespread presence of toxigenic fungi in animal feedstuffs. The absence of statistically significant differences in mycotoxin concentrations between ranched and nomadic goat milk samples is an indicator that farm system does not mitigate mycotoxin occurrence. The mycotoxins detected may be attributed to the ubiquitous contamination of feed materials including hay, cereal crops, and forage by mycotoxigenic fungi. This phenomenon is exacerbated by sub-tropical climatic conditions in Nigeria and sub-Saharan Africa, which promote fungal proliferation and mycotoxin production (Onyeke 2020; Ezekiel et al. 2021; Ukwuru and Muritala 2023).

Milk microbiota may serve as biotechnological agents for mitigating low-dose mycotoxin contamination during the production of fermented dairy products. While prior research has predominantly focused on aflatoxin (AFB_1_/AFM_1_) detoxification, several microbial strains have demonstrated efficacy in mycotoxin binding or enzymatic degradation. For instance, we established in a prior study that multiple strains of *Pichia kudriavzevii* could decontaminate multi-mycotoxin during food fermentation by process of binding and absorption (Ezekiel et al. 2025). In other studies, specific strains of *Streptococcus thermophilus* (Chen et al. 2015) and *Staphylococcus warneri* (Adebo et al. 2016) exhibit strong aflatoxin-binding capacities, while *Saccharomyces cerevisiae* (Chlebicz and Śliżewska 2020) can adsorb AFB_1_, reducing its bioavailability. Enzymatic detoxification has been observed in *Bacillus subtilis* ANSB060 (Gao et al. 2011), *Bacillus licheniformis* CFR1 (Raksha Rao et al. 2017)d *coli* CG1061 (Wang et al. 2019), which degrade or structurally modify aflatoxins, thereby diminishing their toxicity. While the correlations identified in this study between microbial taxa and mycotoxin levels may reflect indirect associations or confounding variables, they provide a foundational framework for targeted strain screening. Future investigations should prioritize in vitro and in vivo validation of candidate isolates (e.g., *Streptococcus*, *Bacillus*, *Saccharomyces*) to elucidate their mechanisms of action and optimize their application in mycotoxin mitigation strategies. Such efforts could enhance food safety in traditional dairy systems while advancing the development of functional fermented products tailored to sub-Saharan African contexts.

## Conclusion

Understanding the microbiota in raw goat milk is essential for making informed policy decisions on breed and farming systems selection for milk quality improvement, both for consumption and the production of fermented foods in developing countries such as Nigeria. This study sheds light on the impact of breed and farming systems on the microbiological diversity and functionality of goat milk towards consumer safety. The findings indicate that while natural, diverse feeding practices associated with nomadic systems enhance microbial richness and potentially beneficial bacteria, variability and potential risks associated with abundance of pathogenic genera should not be downplayed and must be managed. Our findings suggest that bacteria and yeasts in goat milk hold promising potential for mycotoxin decontamination. However, further research is needed to evaluate their effectiveness in decontaminating these mycotoxins. Findings in this study are expected to inform milk production and public health policies, contributing to developing strategies to improve safety of goat milk and its products in Nigeria. In addition, the study provides insights for the potential exploitation of technologically important microbial strains including options for design of mixed cultures to produce safe traditionally fermented dairy products for ensuring food security. Future research should therefore focus on recognizing the relationship between feed and milk microbiota with a focus on optimising feed practices to maximise microbial benefits while minimising risks.

**Table:** 

Isolate name	Isolate code	Accession number
*Limosilactobacillus fermentum*	30m1	OL354445
*Lactiplantibacillus pentosus*	102s4	OL354444
*Weissella confusa*	92m1	OL354443
*Weissella cibaria*	93m3	OL362022
*Pedicoccus pentosaceus*	23d	OL354442
*Pedicoccus acidilactici*	18a	OL354441
*Bacillus thuringiensis*	78f	JBNGET000000000
*Escherichia coli*	9a	JBNGER000000000
*Escherichia coli*	9d	JBNGES000000000
*Escherichia coli*	9b	PV133367.1
*Acinetobacter johnsonii*	84m1	PV202798
*Acinetobacter albensis*	84m3	PV202796
*Acinetobacter baumannii*	86m2	PV202803
*Acinetobacter baumannii*	93v5	PV202801
*Lysinibacillus fusiformis*	18s1	PV133371.1
*Enterococcus hirae*	30a17	JBNGEU000000000
*Enterococcus faecalis*	21b17	PV133369.1
*Enterococcus faecalis*	12h	PV133372.1
*Bacillus sp.*	45m3	PV202804
*Bacillus velezensis*	72m2	PV202809
*Pseudomonas paraeruginosa*	32b17	PV133374.1
*Pseudomonas sp*	87v3	PV202797
*Pseudomonas paralactis*	87m1	PV202799
*Lactobacillus sakei*	45v2	PV202808
*Pseudomonas haemolytica*	79f	PV202806
*Acinetobacter radioresistens*	12s4	PV133366.1
*Staphylococcus condimenti*	88v3	PV202807
*Enterococcus durans*	108a17	PV202810
*Alcaligenes faecalis*	92a17	PV133368.1
*Enterococcus avium*	33a17	PV133373.1
*Lactobacillus sakei*	86m4	JBNGEV000000000
*Morganella morganii*	91m4	JBLURQ000000000.1
*Brevibacillus borstelensis*	90v4	PV202800
*Brevibacillus borstelensis*	90v2	PV202802
*Brevibacillus borstelensis*	23c	JBNGEW000000000
*Providencia rustigianii*	53s3	PV133370.1
*Lactococcus garvieae*	48s3	PV202805
*Candida tropicalis*	12b	OL635644.1
*Candida tropicalis*	108c	OL635642.1
*Candida tropicalis*	20A	OL635640.1
*Candida tropicalis*	65B	OL635639.1
*Candida tropicalis*	20B	OL635638.1
*Candida tropicalis*	12A	OL635636.1
*Candida tropicalis*	33D	OL635635.1
*Kluyveromyces marxianus*	27a	OL635643.1
*Diutina rugosa*	53b	OL635641.1
*Diutina rugosa*	77a	OL635637.1
*Diutina rugosa*	120b	OL635634.1

## Supplementary Information

Below is the link to the electronic supplementary material.

Supplementary Material 1

Supplementary Material 2

Supplementary Material 3

Supplementary Material 4

Supplementary Material 5

Supplementary Material 6

## Data Availability

The obtained single-cell whole genome along with the 16 S and 26 S Illumina-MiSeq sequence datasets have been deposited in GenBank under the BioProject number PRJNA1086807. The reference sequence and taxonomic classification table files used for training a 26 S classifier for this study are openly available in Figshare at https://doi.org/10.6084/m9.figshare.27054943.v1. The 16S and 26S nucleotide sequences generated from individual microbial isolates in this study have been deposited in the NCBI GenBank public sequence repository with assigned accession numbers provided below.
