# Supplementary figures and images for: Functional and safety profiles of microbial communities in milk of ranched and nomadic goats and their predictive role in mycotoxin reduction
Source: World J Microbiol Biotechnol. 2025 Aug 1;41(8):292. doi: 10.1007/s11274-025-04507-3 (PMC12316834; doi:10.1007/s11274-025-04507-3)

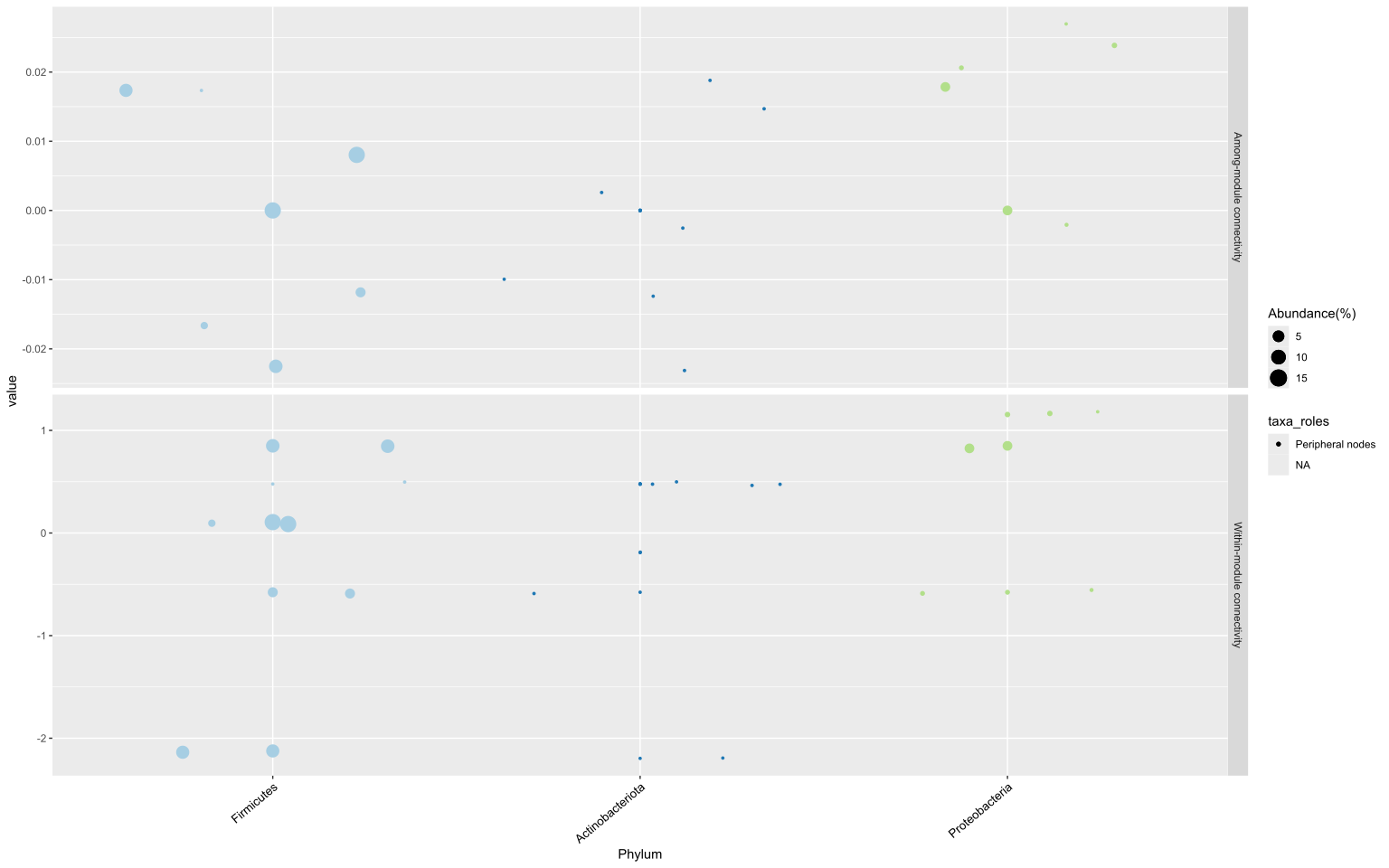

Supplement: Supplementary file 1 — Supplementary Material 1 [file 11274_2025_4507_MOESM1_ESM.png]

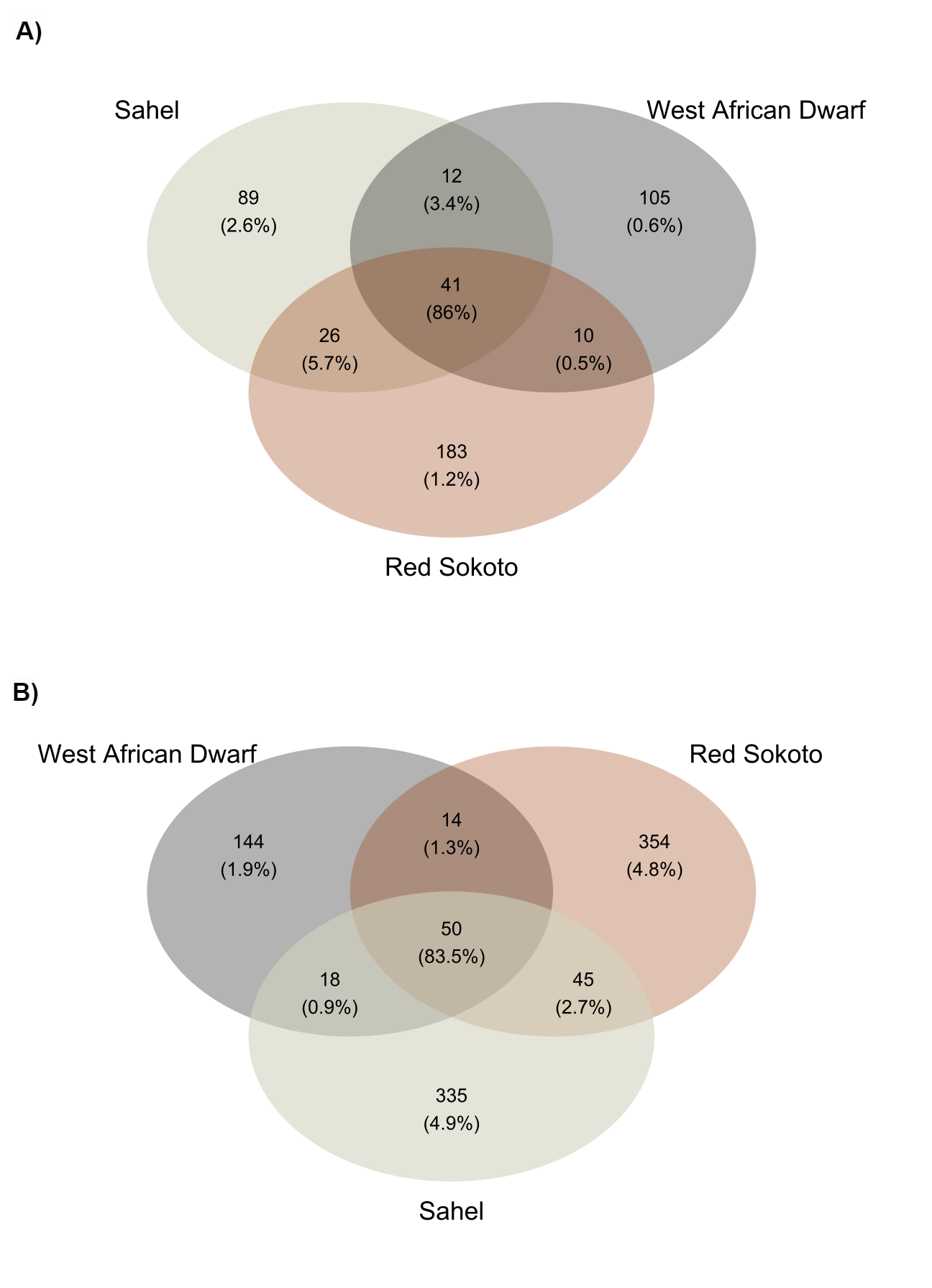

Supplement: Supplementary file 2 — Supplementary Material 2 [file 11274_2025_4507_MOESM2_ESM.png]

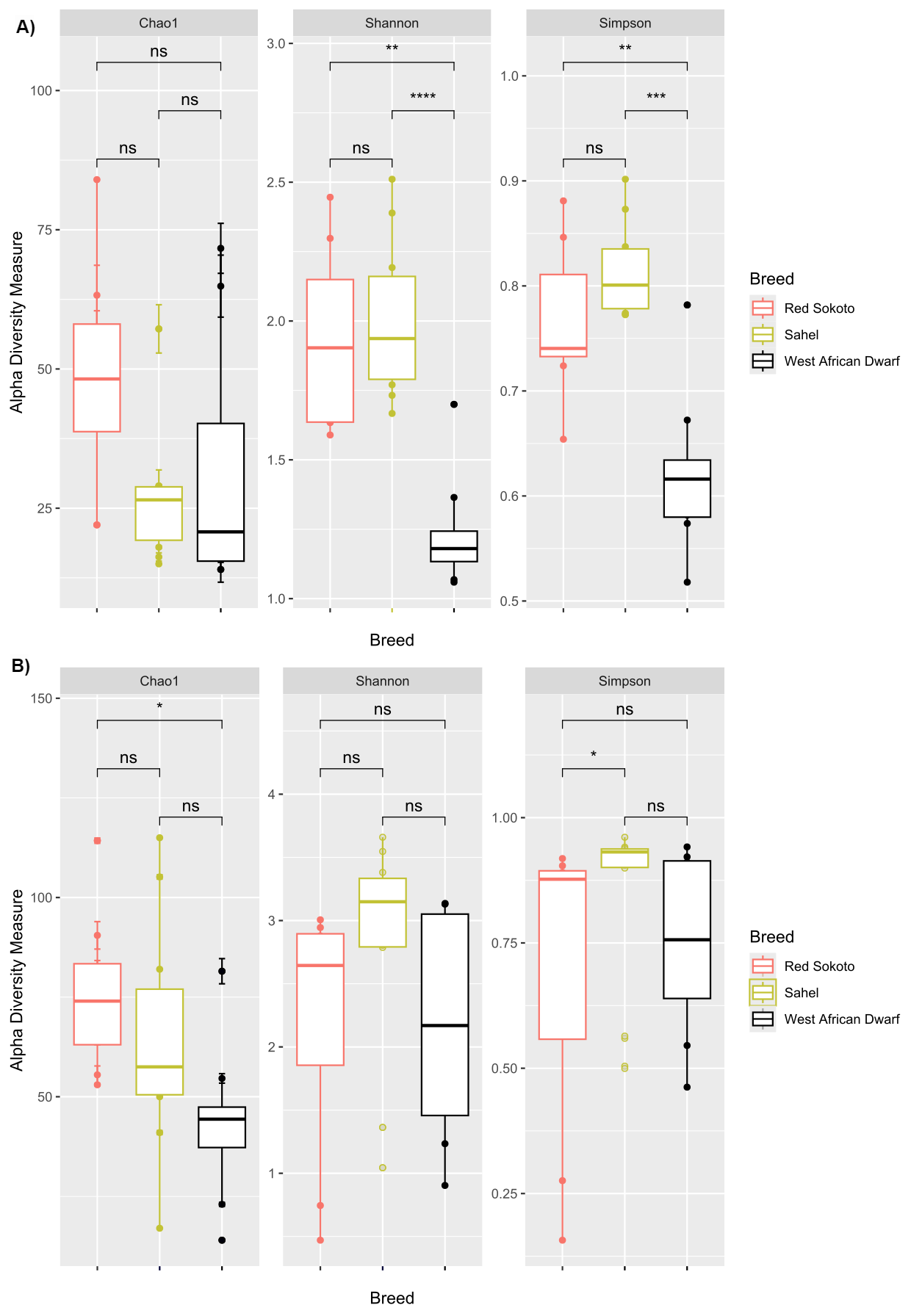

Supplement: Supplementary file 3 — Supplementary Material 3 [file 11274_2025_4507_MOESM3_ESM.png]

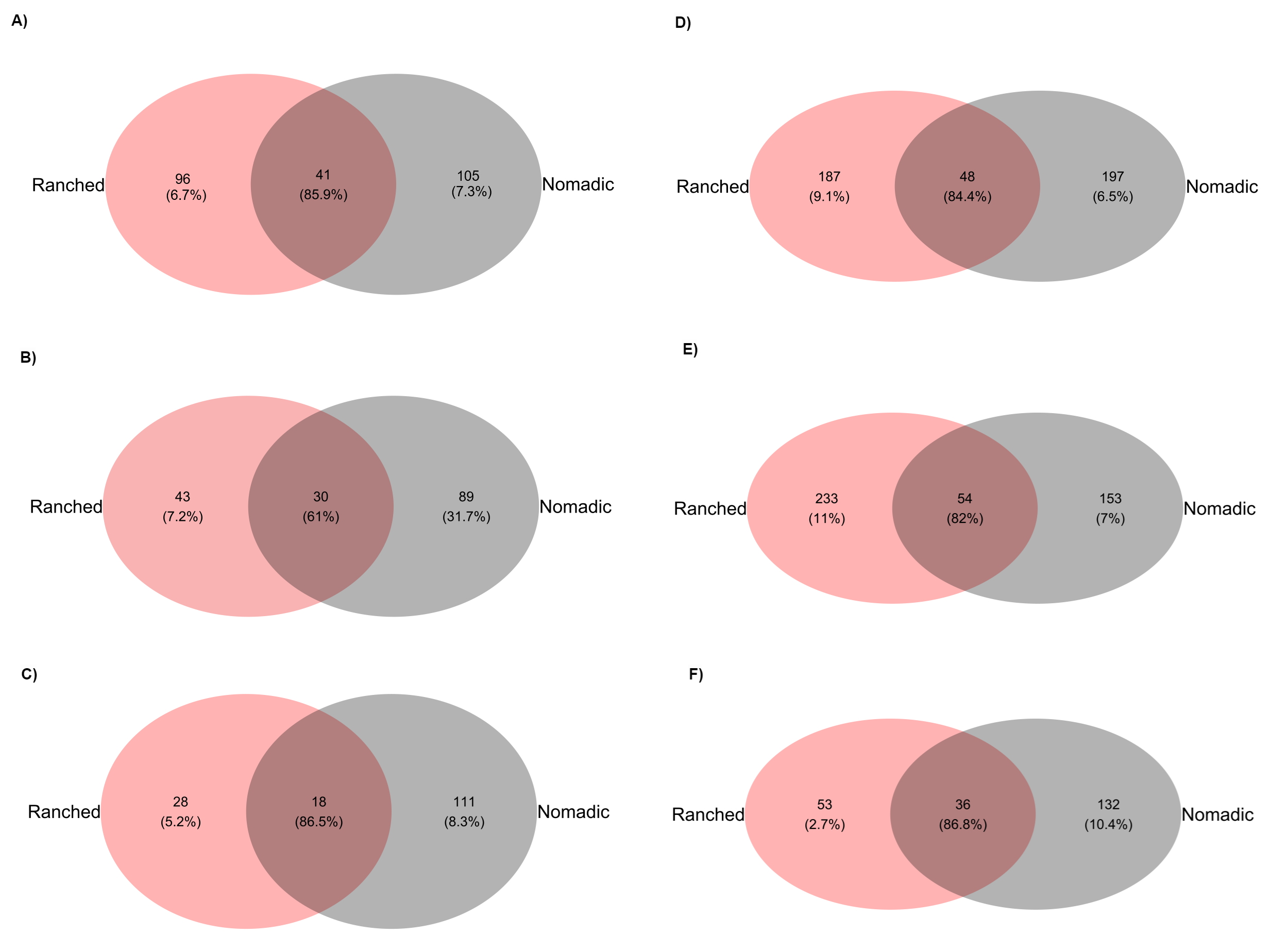

Supplement: Supplementary file 4 — Supplementary Material 4 [file 11274_2025_4507_MOESM4_ESM.png]

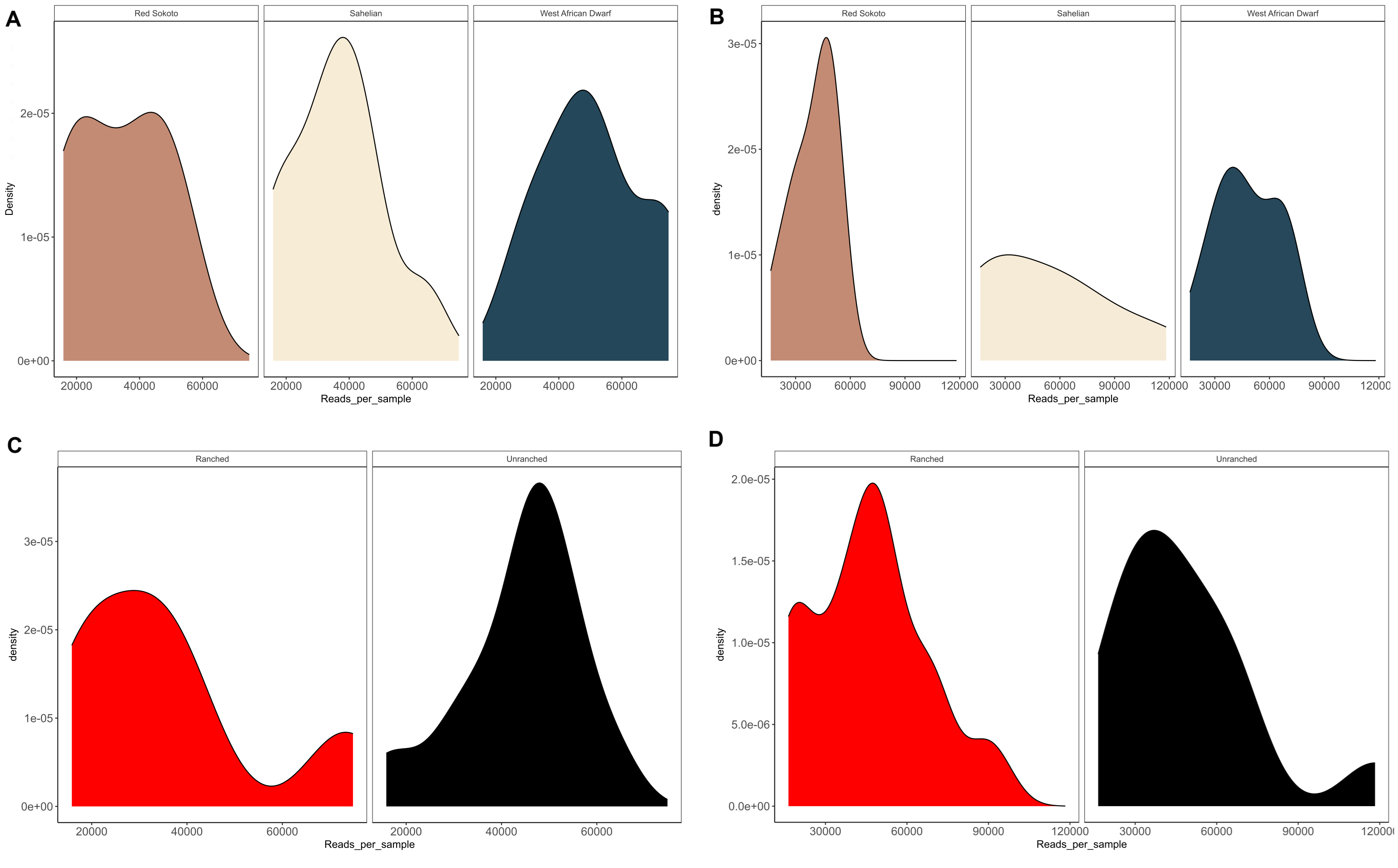

Supplement: Supplementary file 5 — Supplementary Material 5 [file 11274_2025_4507_MOESM5_ESM.tif]

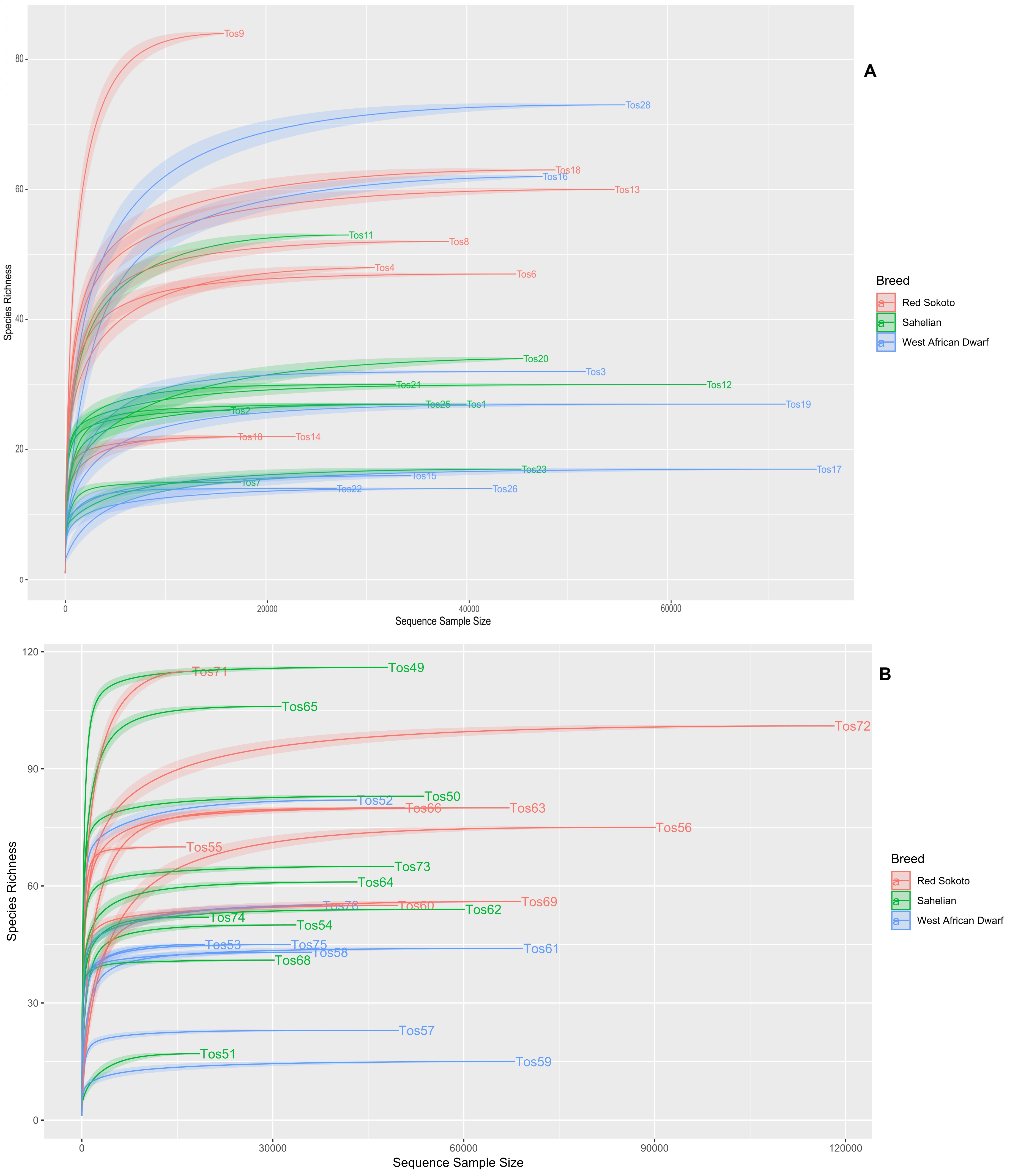

Supplement: Supplementary file 6 — Supplementary Material 6 [file 11274_2025_4507_MOESM6_ESM.tif]
